# The intestinal *Stenotrophomonas maltophilia* ZG-GX enhances the resistance of *Spodoptera frugiperda* to beta-cypermethrin and decreases the amino acid levels

**DOI:** 10.1128/spectrum.03150-25

**Published:** 2025-11-18

**Authors:** Yong Duan, Daozhen Luo, Banghong Jian, Liang Wu, Hao Yang, Chunyu Hu, Yang Zhao

**Affiliations:** 1 State Key Laboratory of Conservation and Utilization of Subtropical Agro-bioresources, Guangxi Key Laboratory of Sugarcane Biology, Guangxi University 12664, Nanning, China; Cleveland Clinic Lerner Research Institute, Cleveland, Ohio, USA

**Keywords:** *Spodoptera frugiperda*, *Stenotrophomonas maltophilia *ZG-GX, beta-cypermethrin

## Abstract

*Spodoptera frugiperda* is an invasive agricultural pest with a high tolerance for chemical insecticides. An increasing number of studies have reported that intestinal microbes of insects can affect host resistance to insecticides. Therefore, in this study, to explore the relationship between *Spodoptera frugiperda* intestinal microbiota and their resistance to pesticides, *Spodoptera frugiperda* was treated with beta-cypermethrin for analysis. The results showed that the abundance of *Stenotrophomonas*, which has the potential to degrade pyrethroid, significantly increased in the intestine. Among them, *Stenotrophomonas maltophilia* ZG-GX was isolated from the intestine. After being fed with ZG-GX, the resistance of *Spodoptera frugiperda* progeny to beta-cypermethrin was significantly increased. In addition, a significant shift occurred in the intestinal microbial community structure and metabolic functions, marked by an increase in the abundance of microbes related to pesticide resistance with ZG-GX treatment. Amino acid levels significantly decreased in *Spodoptera frugiperda* feces after ZG-GX treatment; however, the antioxidant enzyme activities and physiological adaptability remained unchanged. Further, the study revealed that the enhanced resistance of ZG-GX-treated *Spodoptera frugiperda* to beta-cypermethrin might be associated with the capability of ZG-GX to reduce beta-cypermethrin concentration. The study provided an important foundation for a deeper understanding of the resistance mechanisms of *Spodoptera frugiperda* to beta-cypermethrin.

**IMPORTANCE:**

*Spodoptera frugiperda* is an invasive organism that is highly tolerant to chemical pesticides and causes severe losses to agriculture. Studying the mechanism behind which *Spodoptera frugiperda* gains resistance to pesticides is important for agricultural production. Among the mechanisms discussed, the relationship between gut microorganisms of *Spodoptera frugiperda* and their resistance to pesticides is largely elusive. In this study, we demonstrated that *Stenotrophomonas maltophilia* ZG-GX isolated from the intestine of *Spodoptera frugiperda* increased the resistance of *Spodoptera frugiperda* to beta-cypermethrin and has strong potential to degrade beta-cypermethrin. Further, we compared the difference in its mechanism with beta-cypermethrin-induced resistance. It is of great significance for the protection of the ecological environment. The study lays the foundation for a better understanding of the mechanism of insect resistance to pesticides and provides clues for the development of new control strategies.

## INTRODUCTION

*Spodoptera frugiperda* (J.E. Smith) (Lepidoptera: Noctuidae), originally from Latin America, has caused significant damage to agriculture (1). It is a highly migratory invasive species that has expanded to over 70 countries (2). *Spodoptera frugiperda* is well known for its strong adaptability and high tolerance to pesticides, with many chemical pesticides proving ineffective against it (3). Until recently, *Spodoptera frugiperda* has developed resistance to various pesticides, including flubendiamide, chlorantraniliprole, methomyl, thiodicarb, permethrin, chlorpyrifos, deltamethrin, fenvalerate, spinosad, emamectin benzoate, and abamectin (4).

Beta-cypermethrin (C_22_H_19_Cl_2_NO_3_) is a widely used broad-spectrum pyrethroid pesticide, which acts as a contact and stomach poison (5). It is commonly used to control Lepidoptera and Coleoptera (6). Beta-cypermethrin was once considered an effective insecticide against *Spodoptera frugiperda*, but with prolonged and excessive use, *Spodoptera frugiperda* has developed resistance to it. By 2019, the resistance of *Spodoptera frugiperda* to beta-cypermethrin had reached a level 35 times higher than the baseline (i.e., the insecticide resistance ratio at which 50% mortality [RR50] was 35 times higher than the baseline) (4).

The development of insecticide resistance in insects is a complex biological process commonly involving multiple mechanisms: reduced penetration efficiency, effective metabolism, and modification of insecticide targets (7). Reduced penetration efficiency is usually due to a change in the insect body wall structure or the activity of the cytochrome P450 enzyme system, which results in the acceleration of the insecticides’ metabolism. For instance, the reduced cuticular penetration in houseflies, *Aedes aegypti*, *Culex quinquefasciatus*, and *Cx. pipiens pallens* led to their enhanced resistance to conferring clothianidin (8). Zhang et al. (2022) demonstrated that the P450 gene was associated with resistance to thiamethoxam in *Bradysia odoriphaga* (9). In addition, insects can enhance the metabolism of insecticides by increasing the activity of esterases, oxidases, or glutathione transferases, thereby reducing the toxicity of insecticides and developing resistance. For instance, organophosphorus insecticides can be hydrolyzed by esterases and catalyzed by glutathione transferases to bind with glutathione and then excreted from the insect body, while pyrethroid insecticides can be oxidized by oxidases (10). Studies by Tao et al. indicated that GST encoding genes were associated with the resistance of *Anopheles sinensis* to pyrethroid pesticides (11). The modification of insecticide targets involves genetic mutations or alteration of protein expression levels, which hinder the effective binding of insecticides to their biological targets, such as neurotransmitter receptors or ion channels. This results in reduced toxicity and the emergence of resistance. For instance, insects often develop resistance to pyrethroid insecticides due to mutations in their GABA receptors or sodium ion channels (12, 13).

Besides the mechanisms mentioned above, an increasing number of studies indicate that the intestinal microbes of insects can also affect host resistance to insecticides (14). *Wolbachia* was reported to be capable of enhancing the resistance of Drosophila to representative insecticides, including organophosphorus, pyrethroid, and chitin synthesis inhibitors (15). Other researchers have confirmed that bacteria *Pseudomonas* sp. strain CB-2, *Paracoccidioides* TRP, *Sphingomonas* sp. strain Dsp-2, *Fecal Alkaloid-producing Bacteria* (*FAPB*) sp. strain DSP-3, *Streptomyces* sp. strain DT-1, and *Paleobacterium* sp. strain JAS2 can biodegrade pesticides such as chlorpyrifos (16, 17). In addition, the symbiotic bacteria in the intestine of *Bemisia tabaci* were proven to be associated with its resistance to thiamethoxam, imidacloprid, and pyriproxyfen (18). Insect intestinal microbes influence insect drug resistance through various pathways. Firstly, they can break down and metabolize drugs within the host, reducing the effective concentration of the drugs and diminishing their impact. Secondly, these microbes can synthesize metabolic products, such as enzymes and proteins, which promote the host’s metabolism and immune response, enhancing drug tolerance. Moreover, insect intestinal microbes can directly participate in the host’s immune response, altering immune dosages and thereby affecting drug resistance. Although several studies have illustrated the role of intestinal microbes in host resistance to insecticides, the function of intestinal microbes in *Spodoptera frugiperda* resistance to beta-cypermethrin has yet to be demonstrated.

In this study, a strain of *Stenotrophomonas maltophilia* named ZG-GX was isolated from the intestine of *Spodoptera frugiperda* that had been exposed to sublethal doses of beta-cypermethrin. The strain ZG-GX enhanced the resistance of *Spodoptera frugiperda* to beta-cypermethrin. In addition, the study preliminarily explored the capability of *Stenotrophomonas maltophilia* ZG-GX to reduce beta-cypermethrin concentrations, alter the intestinal microbial community structure, and affect the amino acid metabolism of the intestinal microbiota.

## MATERIALS AND METHODS

### Materials

In this experiment, the *Spodoptera frugiperda* was reared in the laboratory for three generations with the corn variety Silver Butterfly Jade 9 (YD9) before the experiments, which had not been treated with any pesticides. The pesticide used was 10% beta-cypermethrin (Syngenta, Basel, Switzerland).

### Isolation of intestinal bacteria

The third-instar larvae of *Spodoptera frugiperda* were stimulated with 150 mg/L of beta-cypermethrin. After 24 h, the surviving *Spodoptera frugiperda* were dissected on an ultra-clean table. The intestine was ground with ddH_2_O. The upper layer of the liquid was applied to LB medium containing 10% beta-cypermethrin (30 mg/mL) and incubated at 37°C for 24 h. A single colony was picked, and the total DNA was extracted using lysozyme. PCR amplification was performed with bacterial universal primers (27F: AGAGTTTGATCCTGGCTCAG; 1492R: GGTTACCTTGTTACGACTT). The products were sent to Sangon Biotech (Shanghai, China) for sequencing services. The selected colonies were sequenced for 16S rRNA, and the results were blasted on NCBI (www.ncbi.nlm.nih.gov/).

### Experimental groups set up

In the experiments, CK1 was set as a blank control without any treatment. CK2 was the progeny of CK1. S1 and S2 were *Spodoptera frugiperda* fed with different concentrations of *Stenotrophomonas maltophilia* ZG-GX bacterial fluids (the optical density at a wavelength of 600 nm was measured as 0.8 for S1 and 1.5 for S2). Pf1 was *Spodoptera frugiperda* treated with 150 mg/L beta-cypermethrin. Pf2 was the progeny larvae of Pf1 and treated with 180 mg/L beta-cypermethrin. Pf3 was the progeny larvae of Pf2 and was treated with 240 mg/L beta-cypermethrin. F was *Spodoptera frugiperda* co-treated with ZG-GX bacterial solution (the optical density at a wavelength of 600 nm was measured as 1.5) and 150 mg/L beta-cypermethrin. Treatments commenced with the third-instar larvae of *Spodoptera frugiperda* and continued until the larvae reached the pupal stage. The experimental procedure involved immersing leaves into 200 mL of a prepared pesticide solution (or bacterial suspension) for approximately 5 s, followed by air-drying before feeding.

In this experiment, a 10% formulation of beta-cypermethrin was used as the stock solution, which was diluted with acetone to obtain the desired concentrations of beta-cypermethrin in a final volume of 200 mL. For the treatment, fresh corn leaves were cut into 1 cm² squares. The prepared corn leaf squares were gradually immersed in the diluted beta-cypermethrin solution for 5 s and then allowed to air dry. These treated leaves were subsequently used to feed *Spodoptera frugiperda* larvae. Initially, one *Spodoptera frugiperda* larva was placed on one piece of the treated corn leaf. When the larvae reached the fifth instar, two pieces of the treated corn leaf were provided for each larva. The mortality and survival rates of the *Spodoptera frugiperda* were recorded 24 h later, and the corn leaves were replaced. This process continued until the larvae entered the pre-pupal stage (indicating cessation of feeding).

### Determination of the toxicological activity of beta-cypermethrin

A preliminary range-finding test with beta-cypermethrin concentrations (0, 100, 200, 400, 800, and 1,600 mg/L; 15 larvae per concentration, three replicates) indicated complete mortality of *Spodoptera frugiperda* larvae at 800–1600 mg/L. Consequently, the formal bioassay focused on the concentration range of 0–800 mg/L to establish the toxicity regression curve. The toxicity of beta-cypermethrin was assessed by feeding third-instar *Spodoptera frugiperda* larvae with leaves dipped in the insecticide solution. Fresh corn leaves (1 cm^2^, 0.02 g) were immersed in a series of beta-cypermethrin solutions (0, 50, 100, 200, 400, and 800 mg/L prepared in acetone) for 5 s and air-dried. For each concentration, 30 larvae (pre-starved for 4 h; individual weight 3–4 mg) were used, with each concentration replicated three times (19). The control group (0 mg/L) received leaves treated with acetone only. The median lethal dose (LD₅₀) was calculated based on the actual amount of pesticide ingested by the larvae, rather than the nominal concentration of the treatment solution. To determine this, the residue concentration of beta-cypermethrin on a per cm² basis of the treated leaves was quantified using high-performance liquid chromatography (HPLC). This measured residue value, combined with the leaf area consumed by the larvae, was used to calculate the actual ingested dose for LD_50_ determination. Toxicity regression equations for the progeny of control groups (CK1, CK2) and treated groups (Pf1, Pf2, S1, S2) were then generated using SPSS software (Ver. 24.0.0.0).

### Determination of the concentration of beta-cypermethrin after ZG-GX treatment

*Stenotrophomonas maltophilia* ZG-GX was cultured in LB medium until a growth density of 0.4 was reached (spectrophotometer at 600 nm). Then, beta-cypermethrin was added to a final concentration of 50 mg/L, and the mixture was cultured in a shaker for seven days (37°C, 200 rpm). Finally, the remaining pesticide was extracted with 10 mL of ethyl acetate. Trace water was removed, and the concentration of beta-cypermethrin was determined by high-performance liquid chromatography (HPLC) (determination method refers to the agricultural industry standard of the People’s Republic of China, NY/T 761-200814, pesticide multi-residue screen methods for determination of organophosphorus pesticides, organochlorine pesticides, pyrethroid pesticides, and carbamate pesticides in vegetables and fruits). The conditions for the determination of beta-cypermethrin by the HPLC were as follows: in the HPLC analysis of beta-cypermethrin, the sample injection temperature was set at 200°C, and the detector temperature was adjusted to 320°C. At the start of the analysis, the column temperature is held at 150°C for 2 min and then increased at a rate of 6°C/min up to 270°C, where it is maintained for 8 min. The carrier gas was N_2_ (≥99.999%) at a flow rate of 1 mL/min. The auxiliary gas was also N_2_ (≥99.999%) at a flow rate of 60 mL/min.

### Antioxidant enzyme activity determination

The activities of the antioxidant enzymes glutathione S-transferase (GST), catalase (CAT), and peroxidase (POD) were measured using commercial assay kits for CAT (A007-2-1), POD (A084-1-1), and GST (A004-1-1), respectively, provided by Jiancheng Biotech (Nanjing, China).

### Anatomy of the intestine of *Spodoptera frugiperda* and microbial sequencing

The fifth-instar larvae of *Spodoptera frugiperda* were used for anatomical analysis of intestine samples using the same method above (repeat three times). The 16S rRNA amplicon sequencing was sent to Gene Denovo (https://www.omicsmart.com) for microbial sequencing.

### Statistical analyses

Statistical analysis was performed on the Omicsmart platform (https://www.omicsmart.com). In this study, we analyzed the relative abundance of *Stenotrophomonas maltophilia* in the gut of *Spodoptera frugiperda* before and after treatment using Krona pie charts. We randomly selected individuals of *Spodoptera frugiperda* for sample collection, dividing them into a control group (before treatment) and an experimental group (after treatment), and extracted gut bacterial DNA. The V3–V4 region of the 16S rRNA gene was amplified using PCR, followed by high-throughput sequencing. We processed the data using the QIIME2 platform to calculate the relative abundance of *Stenotrophomonas maltophilia*. Finally, we organized the data into Krona input format and generated a Krona pie chart to visually represent the impact of treatment on the abundance of *Stenotrophomonas maltophilia*.

The Tukey HSD test was used to compare the changes in gut bacterial abundance or alpha diversity (at the genus level) between the control and the beta-cypermethrin treatment. Principal component analysis (PCA) was used to determine the similarity of bacterial communities between the control and the treatment. Then, the relative abundance of each type was transformed using log_10_ (X + 1) and used to plot the PCA. LDA effect size (LEfSe) analysis was used to explore the differences in the intestinal changes of *Spodoptera frugiperda* after one generation of treatment with beta-cypermethrin and ZG-GX, respectively, using an established LDA score >2.

### Determination of amino acid levels in feces

Corn leaves were soaked in beta-cypermethrin solution with a concentration of 150 mg/L and then air-dried. The treated leaves were then provided to the fifth-instar larvae of *Spodoptera frugiperda*, which had been subjected to starvation for 12 h. After 24 h, the feces of the *Spodoptera frugiperda* larvae were gathered. The contents of 15 amino acids within the feces were subsequently quantified using liquid chromatography.

## RESULTS

### The intestinal symbiotic bacterium *Stenotrophomonas* was associated with the response of *Spodoptera frugiperda* to beta-cypermethrin treatment

**Fig 1:**
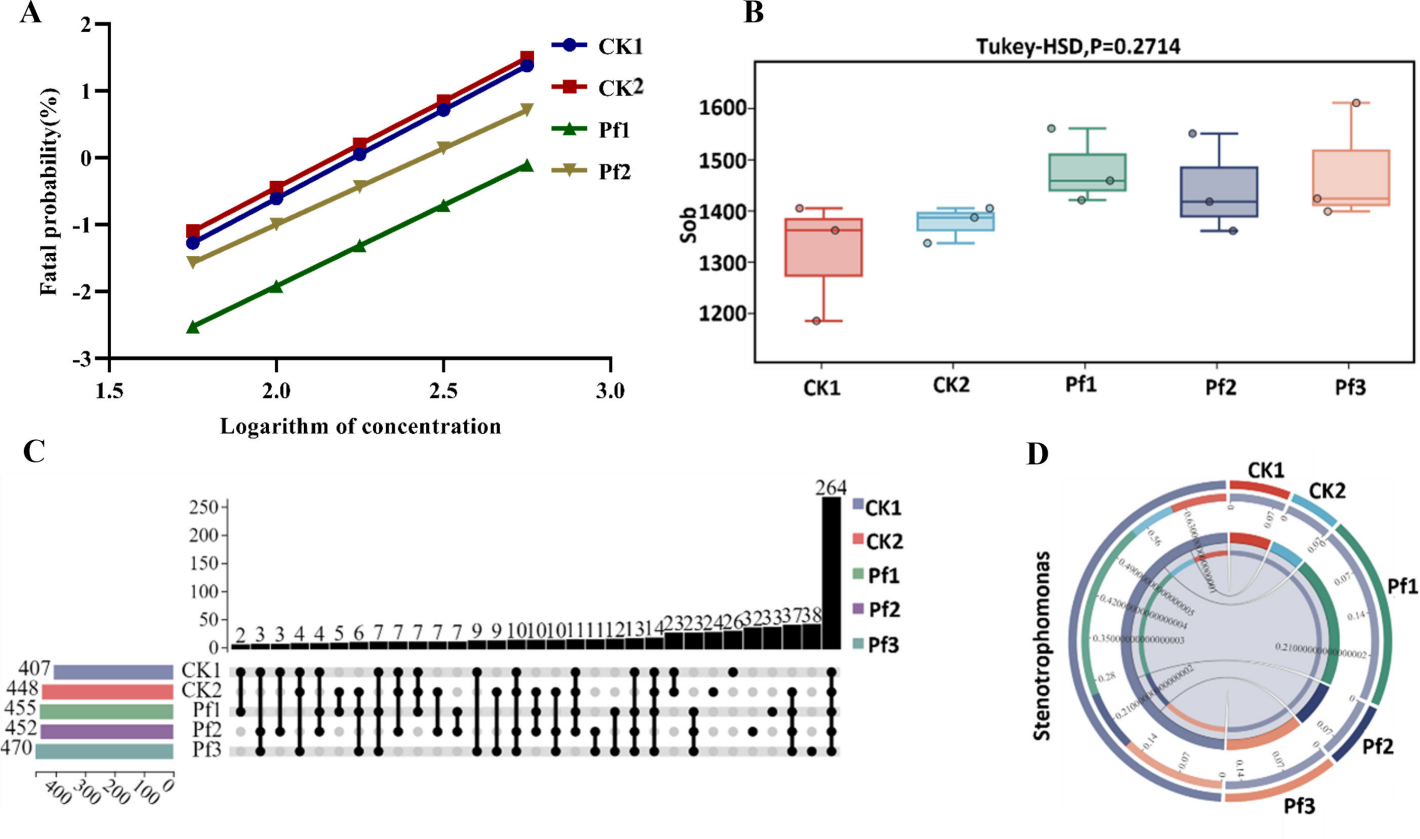
Changes in resistance of *Spodoptera frugiperda* after three successive generations of beta-cypermethrin treatment and changes in intestinal microorganisms of *Spodoptera frugiperda* after beta-cypermethrin treatment. (**A**) Virulence regression equations of beta-cypermethrin continuous treatments for two generations of *Spodoptera frugiperda*. (**B**) Statistical results of Tukey HSD for the structural abundance of intestinal microbial communities (Sob) of intestinal microorganisms in the control group (CK1, CK2) and *Spodoptera frugiperda* after stimulation of beta-cypermethrin (Pf1, Pf2, and Pf3) (*P* = 0.2714). (**C**) Upset plots of *Spodoptera frugiperda* intestinal microbial communities at the level of bacterial genus after beta-cypermethrin stimulation (Pf1, Pf2, and Pf3) compared to controls (CK1, CK2). (**D**) Relative abundance of *Stenotrophomonas* in the intestine of beta-cypermethrin-treated *Spodoptera frugiperda* (Pf1, Pf2, and Pf3) and in the intestine of *Spodoptera frugiperda* (CK1, CK2) not stimulated with pesticides, respectively.

To investigate the relationship between the intestinal microbiota of *Spodoptera frugiperda* and its resistance to beta-cypermethrin, *Spodoptera frugiperda* was continuously treated with the median lethal dose of beta-cypermethrin (LD_50_) for three generations (Pf1, Pf2, Pf3, with increased concentration in accordance with each LD₅₀), and both resistance to beta-cypermethrin and alterations in the intestinal microbiome of *Spodoptera frugiperda* were analyzed. Before treatment, the toxicity virulence regression equation of *Spodoptera frugiperda* (CK1) and its progeny (CK2) to beta-cypermethrin was first determined: Y = −5.91 + 2.65X (*R*² = 0.985) for CK1 and Y = −5.63 + 2.59X (*R*² = 0.991) for CK2 (Fig. 1A). From this, the LD_50_ causing 50% mortality in *Spodoptera frugiperda* larvae (72 h) was determined to be 7.25 mg/kg (95% confidence interval [CI]: 5.84–8.94 mg/kg). The similarity between the toxicity virulence regression equations of CK1 and CK2 suggested that the resistance to beta-cypermethrin across different generations was negligible. The Pf1 group was treated with 150 mg/L beta-cypermethrin, as determined based on above the LD_50_. The toxicity virulence regression equation for Pf1 was Y = −5.51 + 2.42X (*R*² = 0.999), with an LD50 (72 h) of 9.1 mg/kg (95% CI: 6.85–11.27 mg/kg) (Fig. 1A). Pf2 was the progeny larvae of Pf1 and treated with 180 mg/L beta-cypermethrin based on the Pf1 LD_50_. The toxicity virulence regression equation for Pf2 was Y = −5.56 + 2.28X (*R*² = 0.977) with an LD_50_ (72 h) of 12.07 mg/kg (95% CI: 9.8–15.02 mg/kg) (Fig. 1A). Pf3 was the progeny of Pf2 larvae and was treated with 240 mg/L beta-cypermethrin, based on the Pf2 LD_50_ (Table 1). Compared to CK1, both the Pf1 and Pf2 groups had smaller slopes (b values) in the beta-cypermethrin toxicity regression equations and higher LD₅₀ values, which indicated an increase in the resistance of *Spodoptera frugiperda* after exposure to beta-cypermethrin.

**TABLE 1:** Poisoning activity of beta-efficiency cypermethrin against third-instar larvae of *Spodoptera frugiperda* (72 h)^*a*^

Group	df	Virulence regression equation	LD25 (95% Cl)	LD50 (95% Cl)	LD95 (95% Cl)	X^2^
CK1	3	Y = −5.91 + 2.65X (*R*^2^ = 0.985)	4.2 mg/kg (2.66–5.27 mg/kg)	7.25 mg/kg (5.84–8.94 mg/kg)	27.41 mg/kg (19.76–46.25 mg/kg)	1.643
CK2	3	Y = −5.63 + 2.59X (*R*^2^ = 0.991)	3.98 mg/kg (2.86–5.4 mg/kg)	6.96 mg/kg (5.58–9.23 mg/kg)	29.17 mg/kg (20.91–49.82 mg/kg)	1.451
Pf1	3	Y = −5.51 + 2.42X (*R*^2^ = 0.999)	5.22 mg/kg (3.84–6.53 mg/kg)	9.1 mg/kg (6.85–11.27 mg/kg)	35.35 mg/kg (25.25–60.37 mg/kg)	2.457
Pf2	3	Y = −5.56 + 2.28X (*R*^2^ = 0.977)	6.97 mg/kg (5.23–8.65 mg/kg)	12.07 mg/kg (9.8–15.02 mg/kg)	45.98 mg/kg (32.47–80.46 mg/kg)	2.98
S1	3	Y = −5.47 + 2.40X (*R*^2^ = 0.982)	5.32 mg/kg (3.96–6.66 mg/kg)	9.32 mg/kg (7.51–11.53 mg/kg)	36.27 mg/kg (25.86–62.25 mg/kg)	2.662
S2	3	Y = −5.31 + 2.29X (*R*^2^ = 0.990)	5.63 mg/kg (4.51–7.06 mg/kg)	10 mg/kg (8.03–12.42 mg/kg)	39.98 mg/kg (28.16–78.83 mg/kg)	2.448

^*a*^ LD25, LD50, and LD95 are the lethal doses (25%, 50%, and 95%) of beta-cypermethrin for *Spodoptera frugiperda* larvae, estimated from the regression of log concentration against mortality. CL stands for confidence limit, and *R*² is the coefficient of determination for the regression equation.

The intestinal microbiota of fifth-instar larvae of *Spodoptera frugiperda* (CK1, CK2, Pf1, Pf2, and Pf3 groups) were compared using 16S rRNA amplicon sequencing. Sob, ACE index, Chao1 index, Shannon–Weiner index, and Simpson’s index were used to estimate the alpha diversity. The results showed that alterations in the intestinal microbial communities of *Spodoptera frugiperda* larvae subjected to treatment with beta-cypermethrin were not significant (Fig. 1B); however, new microbes appeared in the intestines, including 26 new bacterial genera (Fig. 1C). In addition, a shift in the relative abundance of the inherent microbes occurred. At the genus level, there were 248 common bacterial genera between the beta-cypermethrin-treated groups (Pf1, Pf2, and Pf3) and controls (CK1 and CK2). However, the relative abundance of *Neisseria*, *Burkholderia*, and *Leptospirillum*, among others, was reduced in Pf1, Pf2, and Pf3 compared to CK1 and CK2. Notably, within the top 100 abundant bacterial genera, 16 genera were more abundant in the Pf1, Pf2, and Pf3 than in the CK1 and CK2. These 16 genera can be categorized into two types: bacterial genera related to insecticide resistance, including *Enterobacter*, *Comamonas*, *Sphingobacterium*, *Clostridium sensu stricto 1*, *Stenotrophomonas*, *Delftia*, and *Flavobacterium*, and those not directly related to insecticide resistance, including *Enterococcus*, *Allorhizobium-Neorhizobium-Pararhizobium-Rhizobium*, *Ralstonia*, *Massilia*, *Solibacillus*, *Herbaspirillum*, *Sedimentibacter*, and *Colidextribacter*. Several species within the genus *Stenotrophomonas* have been proven to be capable of degrading cypermethrin. Notably, the relative abundance of *Stenotrophomonas* was significantly changed in the intestine of *Spodoptera frugiperda* larvae after pesticide exposure, suggesting its possible importance in the response of *Spodoptera frugiperda* to beta-cypermethrin (Fig. 1D; Fig. S1).

### *Stenotrophomonas maltophilia* ZG-GX can reduce the concentration of beta-cypermethrin

**Fig 2:**
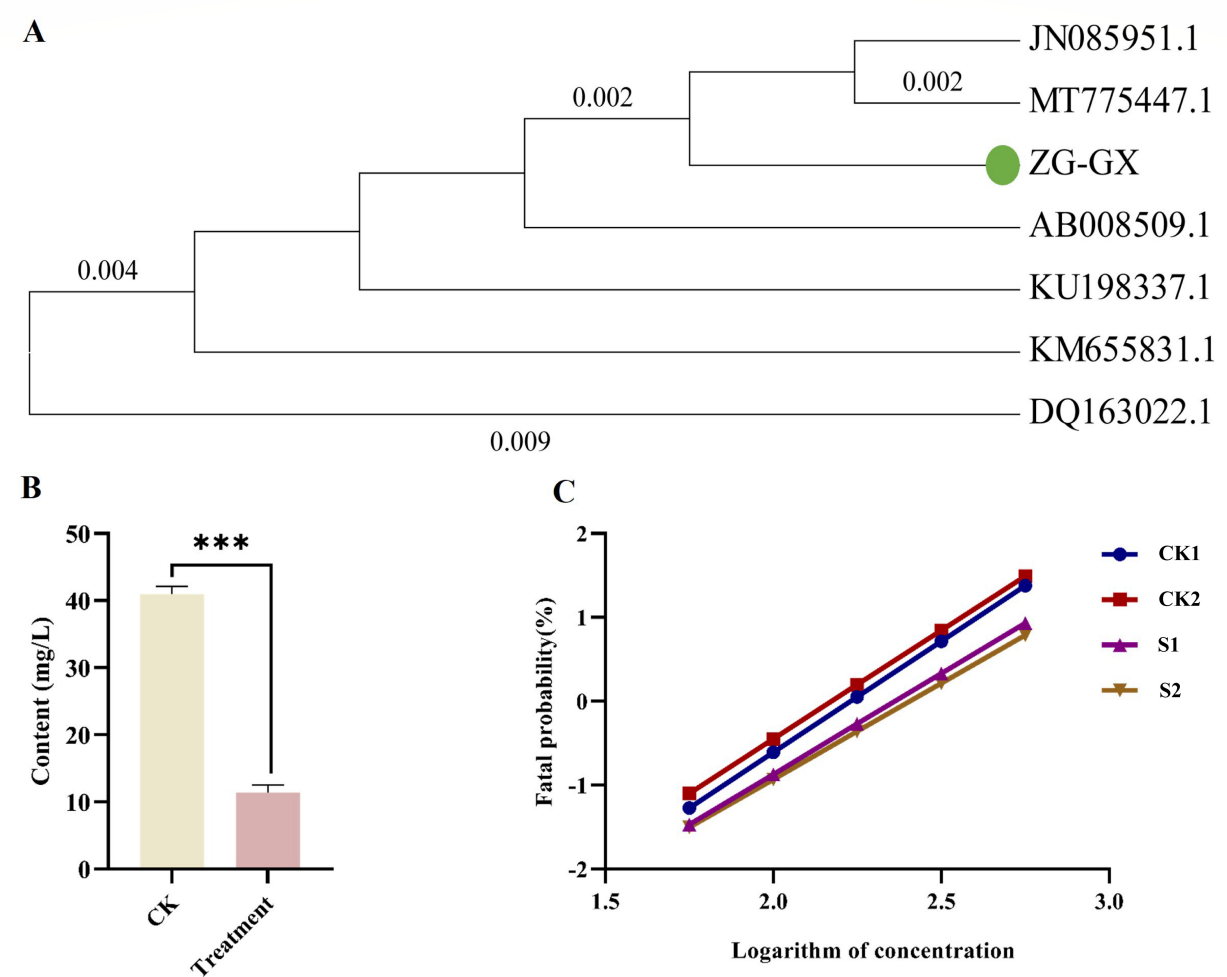
Concentration reduction of beta-cypermethrin by S*tenotrophomonas maltophilia* ZG-GX treatment and enhancement of resistance to beta-cypermethrin in *Spodoptera frugiperda*. (**A**) Phylogenetic tree of ZG-GX isolated from the intestine of *Spodoptera frugiperda*. All other sequences in the phylogenetic tree were bacteria of the genus *Stenotrophomonas*. The bacterial sequences were derived from the GenBank accession numbers that have been labeled after the species name. (**B**) Linear plot of virulence regression equation of toxicity of *Spodoptera frugiperda* in CK1, S1, and S2 groups. ***P ≤ 0.001. (**C**) Linear plot of virulence regression equation between *Stenotrophomonas maltophilia* ZG-GX-fed *Spodoptera frugiperda* (S1, S2) and controls (CK1, CK2). S1 and S2 were fed with *Stenotrophomonas maltophilia* ZG-GX (the optical density at a wavelength of 600 nm was measured as 0.8 [S1] and 1.5 [S2]) bacterial fluids. S was treated with 50 mg/L of beta-cypermethrin, and the concentration of the optical density at a wavelength of 600 nm was measured as 0.4 for seven days. The control (CK) did not receive any bacterial fluid.

To further investigate the relationship between *Stenotrophomonas* and the resistance of *Spodoptera frugiperda* to beta-cypermethrin, intestinal bacteria were isolated from the intestine of *Spodoptera frugiperda*. The selected colonies were sequenced for 16S rRNA. It was found that one strain belonging to the order *Xanthomonadales*, family *Xanthomonadaceae*, and genus *Stenotrophomonas* shared 99.58% sequence similarity with *Stenotrophomonas maltophilia* PVC-SHT (GenBank accession number MK300721.1). This strain was named *Stenotrophomonas maltophilia* ZG-GX (see sequence and comparison chart in the attached figure). To further characterize *Stenotrophomonas maltophilia* ZG-GX, a phylogenetic tree was constructed using sequences of *Stenotrophomonas* strains clearly identified in the literature as capable of degrading pesticides. These sequences included *Stenotrophomonas maltophilia* ZL1, which can degrade 17β-estradiol (20); *Stenotrophomonas maltophilia* D310-3, which can degrade chlorimuron-sulfuron (21); *Stenotrophomonas maltophilia* XQ08, capable of degrading deltamethrin in effluent soils and vegetables (22); *Stenotrophomonas maltophilia* ZJB-14120, capable of degrading avermectin (23); and *Stenotrophomonas maltophilia* OK-5, capable of degrading TNT (24). The results showed that *Stenotrophomonas maltophilia* ZG-GX had a higher bootstrap value compared to other pesticide-degrading *Stenotrophomonas* (Fig. 2A), suggesting that ZG-GX may have the potential to degrade beta-cypermethrin.

To further investigate whether *Stenotrophomonas maltophilia* ZG-GX can reduce beta-cypermethrin concentration, a solution of *Stenotrophomonas maltophilia* ZG-GX was spiked with 50 mg/L of beta-cypermethrin (initial concentration). The treatment and the control group without *Stenotrophomonas maltophilia* ZG-GX added were cultured under the same conditions for seven days. The residual beta-cypermethrin concentrations were determined by liquid chromatography. The results showed that the concentration of beta-cypermethrin in the treatment group containing *Stenotrophomonas maltophilia* ZG-GX decreased to 10.71 mg/L, while the concentration in the control group was 42.618 mg/L. Thus, it was concluded that ZG-GX was able to significantly reduce the concentration of beta-cypermethrin when coexisting with beta-cypermethrin (Fig. 2B).

### *Stenotrophomonas maltophilia* ZG-GX can enhance the resistance of *Spodoptera frugiperda* to beta-cypermethrin

To further investigate whether ZG-GX can influence the resistance of *Spodoptera frugiperda* to beta-cypermethrin, the dipping leaf method was employed to apply the isolated *Stenotrophomonas maltophilia* ZG-GX to *Spodoptera frugiperda* directly. Considering the potential concentration effect of ZG-GX on *Spodoptera frugiperda*, two concentration treatments were set up and compared: S1 (OD_600_ = 0.8) and S2 (OD_600_ = 1.5), respectively. The resistance of the progeny larvae of *Spodoptera frugiperda* in groups S1 and S2 to beta-cypermethrin was determined, with the toxicity virulence regression equations for S1 and S2 being Y = −5.47 + 2.40X (*R*^2^ = 0.982) and Y = −5.31 + 2.29X (*R*^2^ = 0.990), respectively (Fig. 2C). Furthermore, the LD_50_ values for S1 and S2 were 9.32 mg/kg (95% CI: 7.51–11.53 mg/kg) and 10.0 mg/kg (95% CI: 8.03–12.42 mg/kg), respectively. Compared to CK1, the toxicity virulence regression equation slopes (b values) were significantly smaller in both the S1 and S2 groups, with higher LD_50_ values. Compared to the LD_50_ of CK1 (7.25 mg/kg; 95% CI: 5.84–8.94 mg/kg), the treatment with ZG-GX significantly increased (*P* < 0.05) the resistance of *Spodoptera frugiperda* to beta-cypermethrin, with the LD_50_ values of S1 and S2 being 1.29 times and 1.38 times that of CK1, respectively. This indicated that *Stenotrophomonas maltophilia* ZG-GX enhanced the resistance of *Spodoptera frugiperda* to beta-cypermethrin.

### Physiological effects of ZG-GX on *Spodoptera frugiperda*

**Fig 3:**
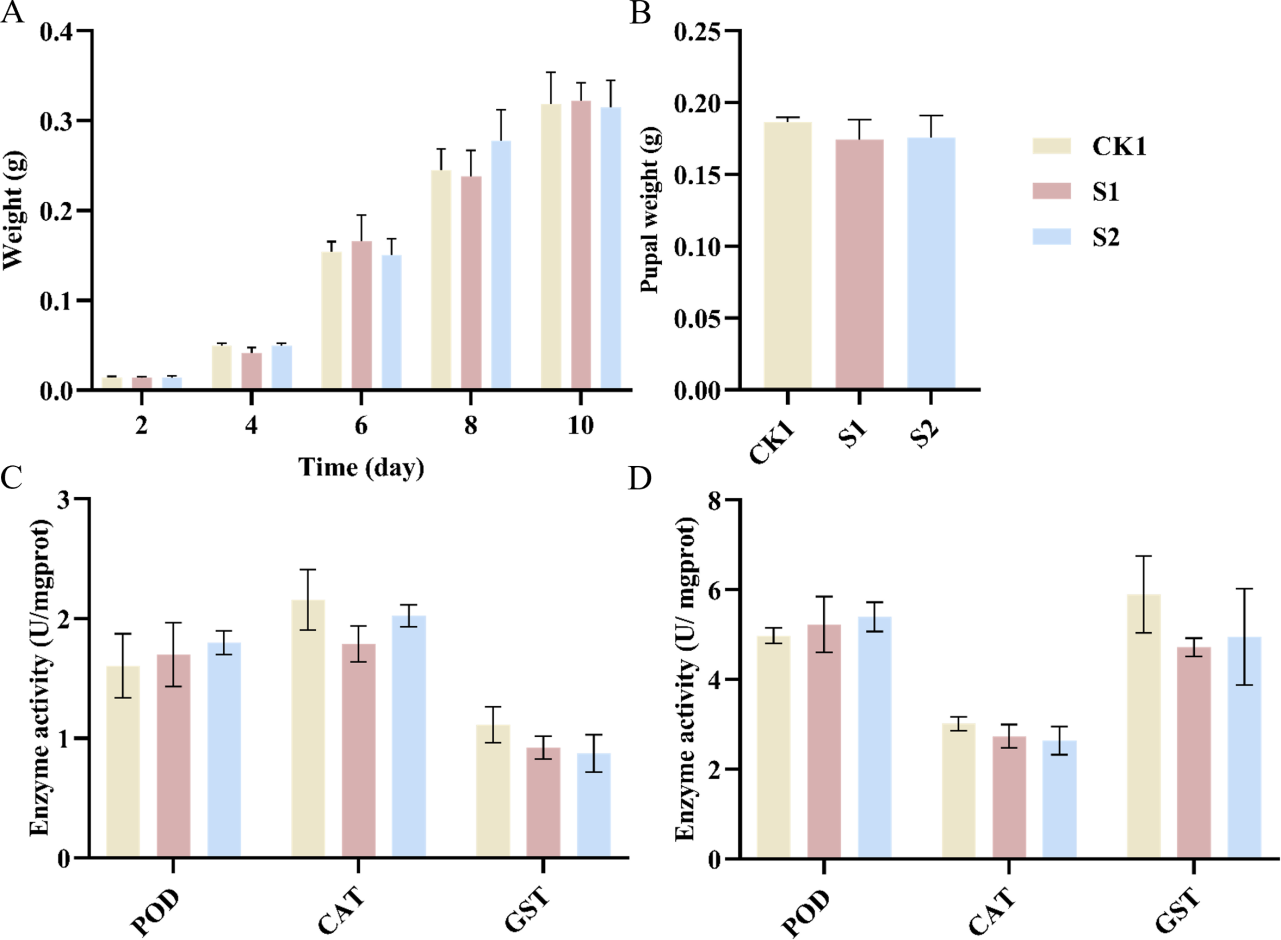
Histograms of antioxidant enzyme activity, body weight, and pupal weight of *Spodoptera frugiperda* after feeding with S*tenotrophomonas maltophilia* ZG-GX. (**A**) Histogram of body weight of *Spodoptera frugiperda* larvae at various stages of growth. (**B**) Histogram of the pupal weight of *Spodoptera frugiperda* after 24 h of pupation. (**C**) Histogram of GST, CAT, and POD enzyme activity of *Spodoptera frugiperda* in S1, S2, and CK1. (**D**) Histograms of GST, CAT, and POD antioxidant enzyme activities of *Spodoptera frugiperda* in S1, S2, and CK1 after 24 h of stimulation with 150 mg/L beta-cypermethrin.

To investigate whether the impact of ZG-GX on the resistance of *Spodoptera frugiperda* to beta-cypermethrin was accompanied by changes in antioxidant enzyme activity and physiological adaptability, the growth index (weight change during the period of the treatment with ZG-GX) and several antioxidant enzyme activities (GST, CAT, and POD) of *Spodoptera frugiperda* in groups CK1, S1, and S2 were measured. The results showed no significant differences in the weight change trends of *Spodoptera frugiperda* and the pupal weight 24 h after pupation in the S1 and S2 groups treated with ZG-GX (*P* > 0.05) (Fig. 3A and B). In addition, no significant differences were identified in the activity of enzymes, including GST, CAT, and POD, under conditions with or without pesticide stimulation (*P* > 0.05) (Fig. 3C and D). Collectively, treatment with *Stenotrophomonas maltophilia* ZG-GX had no significant effect on the physiological adaptability of *Spodoptera frugiperda* or on the activity of antioxidant enzymes, whether with or without pesticide stress.

### ZG-GX treatment changed the diversity of the intestinal microbes of *Spodoptera frugiperda*

**Fig 4:**
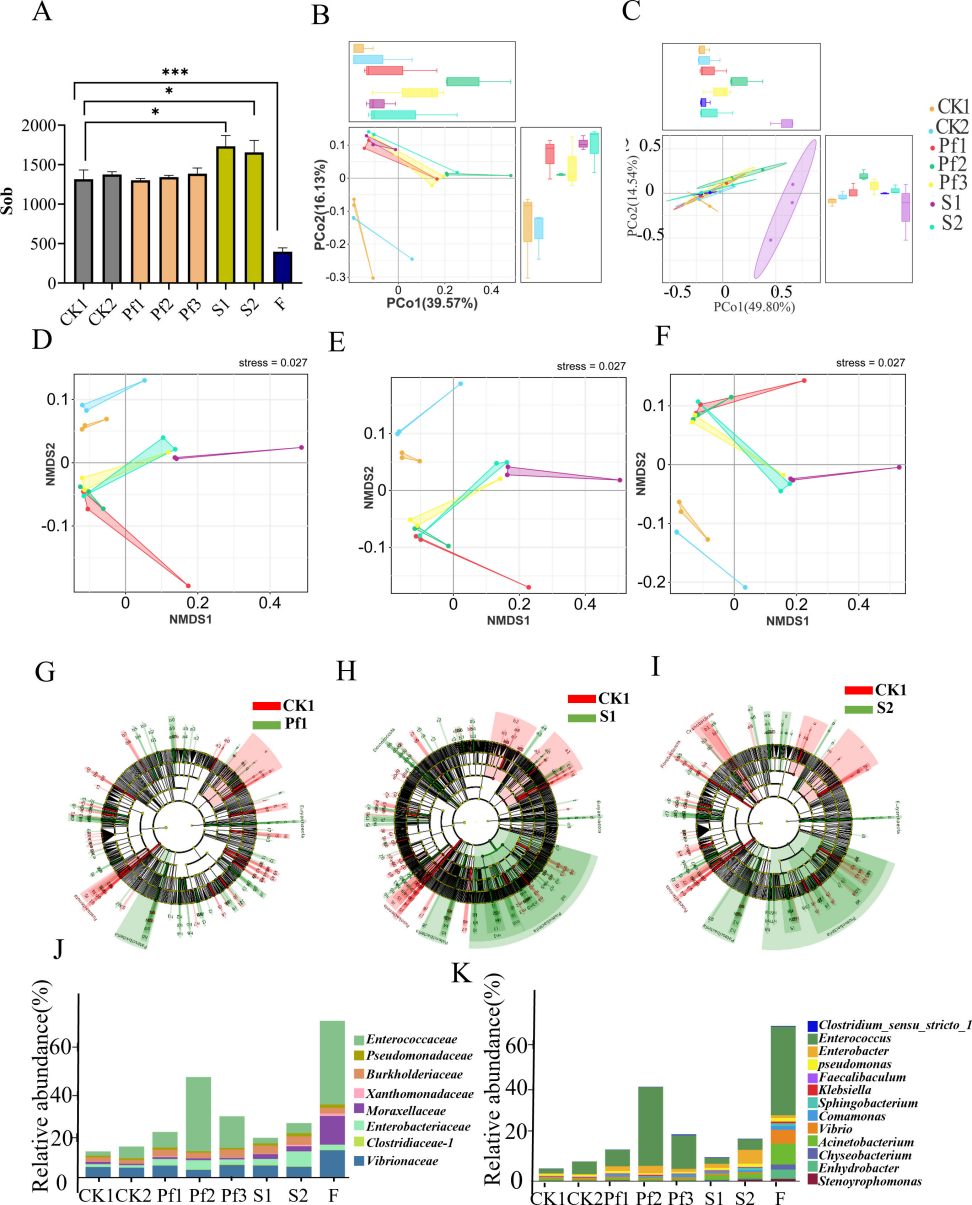
Effects of ZG-GX and beta-cypermethrin on the intestinal microbial structure of *Spodoptera frugiperda*, including overall changes in intestinal microbial structure and changes at the family and genus levels. (**A**) Histogram of sob of the sequencing results of the intestinal microorganisms. (**B**) PCo clustering plot of intestinal microbial OTU values (with group F). (**C**) PCo clustering plot of intestinal microbial OTU values (without group F). (**D–F**) NMDS clustering of intestinal microbial richness at the order, family, and genus level. (**G–I**) Comparative analysis of intestinal microbial community structure using LEfSe: beta-cypermethrin treated (Pf1) and *Stenotrophomonas maltophilia* ZG-GX-fed *Spodoptera frugiperda* (S1, S2) compared to control (CK1). There is a high degree of similarity between (**H**) and (**I**), while (**G**) is more dissimilar to (**H**) and (**I**). There was a high degree of similarity in intestinal microbial structure between ZG-GX-fed *Spodoptera frugiperda* (S1, S2) and a high degree of variability in intestinal microbial structure between ZG-GX-fed *Spodoptera frugiperda* (S1, S2) and beta-cypermethrin stimulated *Spodoptera frugiperda* (Pf1). (**J and K**) Histograms of family and genus with increased relative abundance associated with pesticide metabolic resistance and degradation of cypermethrin in the intestine. CK1 and CK2 were blank control groups without any treatment. Pf1, Pf2, and Pf3 were treated with pesticides. S1 and S2 were fed with ZG-GX. F was the co-treatment of *Spodoptera frugiperda* with ZG-GX bacterial solution (1.5) and 150 mg/L beta-cypermethrin. Significance markers: *P* ≥ 0.05, no marker (ns); *0.01 < *P* < 0.05 markers; **0.001 < *P* ≤ 0.01 markers; ***P ≤ 0.001 markers. The legends for **E, F, and G** are the same as in **A**.

To further investigate and compare the impact of ZG-GX treatment and beta-cypermethrin exposure on the changes in the intestinal microbial structure of *Spodoptera frugiperda*, we conducted a comparative analysis of the intestinal microbes of *Spodoptera frugiperda* from different treatments (CK1, CK2, Pf1, Pf2, Pf3, S1, S2, and F, the co-treatment with ZG-GX and beta-cypermethrin). Indices, including Sob, Chao1, ACE, Shannon, and Simpson, were analyzed as measures of alpha diversity. A significant increase (*P* < 0.05) in the diversity of the intestinal microbial community was observed for the ZG-GX treatment compared to CK1 and CK2. In addition, a significant decrease (*P* < 0.001) in the diversity of the intestinal microbial community was noted for the combined treatment of ZG-GX and beta-cypermethrin (F group) (Fig. 4A).

Principal coordinates analysis (PCoA) of the relative abundance of the major intestinal microbial community revealed that significant differences existed between co-treatment F group and other groups with OTUs (operational taxonomic units) (PCo1: 49.80%, PCo2: 14.54%) (Fig. 4B; the results of the comparison with Group F at the level of the order, genus were shown in Fig. S2). Therefore, to better compare the impact of ZG-GX treatment and beta-cypermethrin on the intestinal microbial community of *Spodoptera frugiperda*, a further PCoA without the F group was conducted at the OTU, bacterial order, family, and genus levels. The results showed that both ZG-GX treatment and beta-cypermethrin treatment were significantly different with the controls based on OTUs (PCo1: 39.57%, PCo2: 16.13%), as well as at the order level (PCo1: 64.25%, PCo2: 13.51%), family level (PCo1: 63.71%, PCo2: 14.67%), and genus level (PCo1: 61.09%, PCo2: 16.03%), respectively (Fig. 4C through F).

LDA effect size (LEfSe) analysis was further performed on *Spodoptera frugiperda* treated with beta-cypermethrin for one generation (Pf1) and those treated with ZG-GX for one generation (S1 and S2). The results showed significant differences in the changes in the intestinal microbiota between the Pf1 and S groups (LDA score >2) (Fig. 4G through I). This indicated that after treatment with beta-cypermethrin and ZG-GX, significant changes occurred in the abundance of related bacteria within the intestinal microbial community of *Spodoptera frugiperda*, potentially affecting its resistance in different ways.

To further investigate the bacteria that might play a role in the enhancement of resistance to beta-cypermethrin in *Spodoptera frugiperda*, the top 60 bacteria in terms of richness content were determined for each treatment group. The bacteria found relevant to pesticide degradation included eight bacterial families—*Enterobacteriaceae*, *Pseudomonadaceae*, *Burkholderiaceae*, *Xanthomonadaceae*, *Moraxellaceae*, *Enterobacteriaceae*, *Clostridiaceae_1*, *Vibrionaceae*—and ten bacterial genera: *Enterococcus*, *Enterobacter*, *Pseudomonas*, *Faecalibaculum*, *Klebsiella*, *Clostridium sensu stricto 1*, *Sphingobacterium*, *Comamonas*, *Vibrio*, and *Stenotrophomonas*. Compared to the control group, the relative abundance of bacterial families such as *Enterobacteriaceae*, *Burkholderiaceae*, and *Xanthomonadaceae*, as well as genera such as *Enterobacter* and *Comamonas*, significantly increased (*P* < 0.05) in the intestine of *Spodoptera frugiperda* treated with ZG-GX, showing distinct differences (Fig. 4J and K; Tables S3 and S4). Comparatively, the significant increase in relative abundance after treatment with beta-cypermethrin was observed in bacterial families such as *Enterococcaceae* and *Vibrionaceae*, and in genera such as *Klebsiella*, *Vibrio*, *Enterococcus*, and *Pseudomonas* (Fig. 4J and K; Tables S3 and S4). Notably, the combined treatment of ZG-GX and beta-cypermethrin (F group) led to a higher relative abundance of these bacteria relevant to pesticide degradation in the *Spodoptera frugiperda* intestine compared to individual treatments, particularly for the *Moraxellaceae*, *Vibrionaceae*, and *Xanthomonadaceae*, and genera such as *Enterococcus*, *Cupriavidus*, and *Pseudomonas* (Fig. 4J and K). This finding suggested that both the ZG-GX treatment and beta-cypermethrin increased the abundance of bacteria relevant to pesticide degradation, with the combined treatment group exhibiting a more pronounced increase.

### Effects of ZG-GX on amino acid metabolism in *Spodoptera frugiperda*

**Fig 5:**
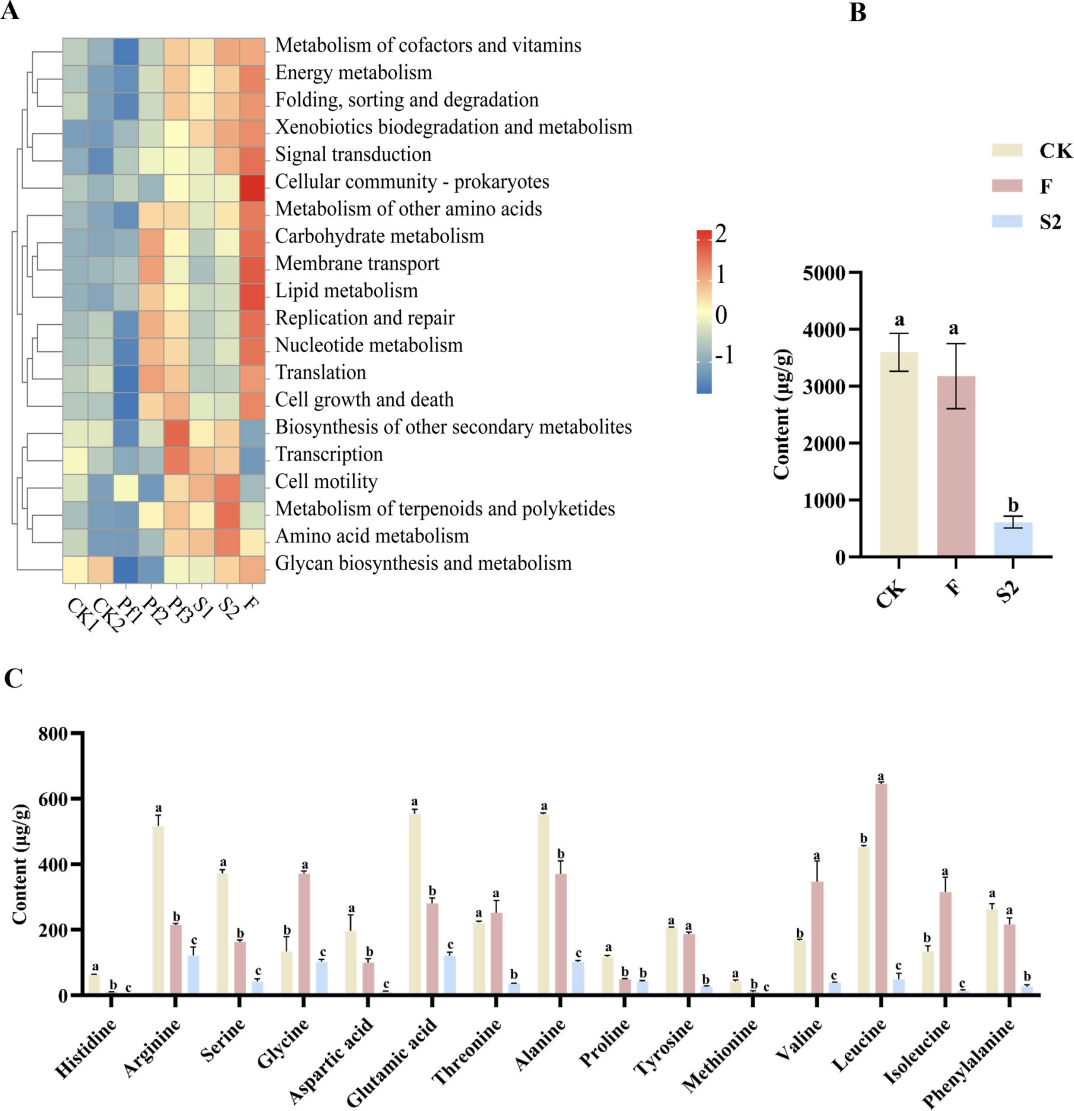
Microbial functional heatmap and amino acid content bar chart. (**A**) Heat map for predicting the function of the intestine microbiome of *Spodoptera frugiperda*. (**B**) Histogram of the total number of the 15 amino acids measured in the feces of *Spodoptera frugiperda*. (**C**) Histogram of the total content of 15 amino acids measured in the feces of *Spodoptera frugiperda*.

The 16S rRNA amplicon sequencing results of the intestinal microbiome of *Spodoptera frugiperda* were further used to predict the metabolic functions of the intestinal microbiota with PICRUSt 2. Among the top 20 major biological functions annotated, the intestinal microbiome of *Spodoptera frugiperda* in the ZG-GX treatment group exhibited significant enhancement in 11 types of metabolic functions (*P* < 0.05) (Fig. 5A). Among these biological functions, those related to insect resistance included the metabolism of cofactors and vitamins, folding, sorting and degradation, xenobiotics biodegradation, and amino acid metabolism. In addition, the combined treatment group F showed an increase in 16 of the top 20 primary biological functions (Fig. 5A). Moreover, the amino acid metabolism of the intestinal microbiome in the ZG-GX treatment group was significantly stronger than that in the beta-cypermethrin treatment group (*P* < 0.05). This suggested that amino acid metabolism might play a critical role in ZG-GX-treated *Spodoptera frugiperda*.

To explore the difference among the amino acid content in feces of *Spodoptera frugiperda* of different treatment groups, the content of 15 amino acids in the feces of fifth-instar larvae of the control, ZG-GX treatment, and F treatment group was measured. The results showed that the total content of amino acids in the feces of the ZG-GX treatment group was significantly reduced (*P* < 0.05), while there was no significant difference in the F group (*P* > 0.05) (Fig. 5B). In addition, for single amino acids, the content of most amino acids in the S2 group (ZG-GX treatment) was significantly lower than that in the control and F groups (*P* < 0.05). Histidine was the amino acid with the most significant change, with its content not detected in the ZG-GX treatment group (Fig. 5C). The F group exhibited significantly decreased amino acid content for histidine, serine, arginine, glutamic acid, and proline (*P* < 0.05) (Fig. 5C), and significantly increased content of glycine, valine, leucine, and isoleucine (*P* < 0.05) (Fig. 5C). The differences in amino acid content between the ZG-GX treatment and the F groups may be due to the addition of beta-cypermethrin in the F group. The above results indicated that ZG-GX treatment significantly altered the amino acid metabolism of *Spodoptera frugiperda*.

## DISCUSSION

### *Stenotrophomonas* is associated with *Spodoptera frugiperda* resistance to beta-cypermethrin

Microorganisms can enhance insect host resistance to pesticides; for instance, the bacterium *Wolbachia* has been proven to increase resistance to imidacloprid in brown planthopper (*Laodelphax striatellus*) by enhancing the expression of genes related to cytochrome P450 (25). Studies have demonstrated that *Stenotrophomonas maltophilia* was associated with insect resistance to a wide range of substances and pesticides by degradation (20–24). It is generally considered that the microbial degradation of pyrethroid insecticides mainly involves ester hydrolysis and oxidation reactions. *Stenotrophomonas maltophilia* XQ08 can ultimately degrade alpha-cypermethrin into small molecules through a variety of reactions (22), and the molecular structure of alpha-cypermethrin is very similar to beta-cypermethrin. Genes related to pesticide degradation have been found in the genome of *Stenotrophomonas*, such as *Stenotrophomonas maltophilia* D457R, which is associated with antibiotics and heavy metals (26). Our study has demonstrated that increased resistance of *Spodoptera frugiperda* to beta-cypermethrin was accompanied by an increase in the relative abundance of *Xanthomonas* and *Stenotrophomonas*. This suggests that *Stenotrophomonas* might be closely related to *Spodoptera frugiperda* resistance.

The b-value in the virulence regression equation of toxicity can be used as a measure of a pesticide’s toxicity to a particular pest or natural enemy (27). The larger the b-value, the more toxic the pesticide is to the pest or natural enemy. In this study, *Stenotrophomonas maltophilia* ZG-GX was able to cause a higher LD_50_ (72 h) and a smaller b-value for beta-cypermethrin in *Spodoptera frugiperda*. Therefore, ZG-GX enhanced the resistance of *Spodoptera frugiperda* to beta-cypermethrin. During this study of resistance, we analyzed no more than three generations, which may reduce the statistical power and limit the accuracy of concluding that resistance has already been obtained. It will be necessary to analyze more generations in future research. Our study also demonstrated that *Stenotrophomonas maltophilia* ZG-GX could reduce the concentration of beta-cypermethrin, suggesting it might be capable of degrading the beta-cypermethrin. We did not further determine the metabolic products after co-cultivation of ZG-GX with beta-cypermethrin, nor did we verify whether antibiotic resistance disappeared after using antibiotics to eliminate bacteria. However, ZG-GX is closely related in the evolutionary tree to various other *Stenotrophomonas maltophilia* capable of degrading pesticides. In particular, *Stenotrophomonas maltophilia* ZG-GX is closely related to *Stenotrophomonas maltophilia* XQ08, which can degrade alpha-cypermethrin that shares the identical chemical bonds with beta-cypermethrin (Fig. S3). Since ZG-GX can reduce the concentration of beta-cypermethrin, it is highly suspected that it may degrade the beta-cypermethrin, which needs further investigation.

### ZG-GX caused changes in the structure of the intestinal microbiota of *Spodoptera frugiperda*, especially the abundance of insecticides-degrading bacteria

In recent years, more and more studies have pointed out that intestinal microorganisms have a non-negligible impact on insect resistance. Besides the direct degradation of pesticides, alterations in the intestinal microbial community of insects can significantly affect their hosts’ resistance. These microorganisms may regulate the host’s detoxification metabolic pathways and enhance its resistance to pesticides. In our study, both pesticide stimulation and *Stenotrophomonas maltophilia* ZG-GX feeding increased the resistance of *Spodoptera frugiperda* to beta-cypermethrin and changed the intestinal microbial community structure. Complex interactions exist between intestinal microorganisms, which further influence and shape the structure of microbial communities within the insect intestine.

Moreover, our study showed that ZG-GX caused changes in the abundance of pesticide-degrading bacteria, including *Enterobacteriaceae*, *Burkholderiaceae*, *Xanthomonadaceae*, *Pseudomonadaceae*, *Comamonadaceae*, and *Haemomonas*. Some studies have already shown that these insect symbiotic bacteria are related to the enhancement of host resistance to pesticides through degradation. For instance, *Pseudomonas aeruginosa* (*Pseudomonadaceae*) can degrade urea in plants by synthesizing deaminase, which reduces the toxic effects of pesticides on insects, such as ciprofloxacin (28). *Bacillus subtilis BSF01*, a bacterium from *Enterobacteriaceae*, can degrade the pyrethroid pesticide cypermethrin through the production of related metabolizing enzymes (29). *Burkholderia cenocepacia* CEIB S5-2 was able to effectively hydrolyze MP and biodegrade p-nitrophenol (PNP), with PNP as the main hydrolysis product (30). *Enterobacter* (*Enterobacteriaceae*) has a certain degradation capacity for beta-cypermethrin and can also use beta-cypermethrin as the only carbon source for growth (31). *Comamonas testosterone* DB-714 (*Comamonadaceae*) has a degradation efficiency of more than 99% for dimethyl phthalate (DMP) (32). The increased abundance of these pesticide-degrading bacteria in the intestine of *Spodoptera frugiperda* may have enhanced the degradation of beta-cypermethrin.

### Antioxidant enzyme activity was not impacted by ZG-GX treatment

Intestinal microorganisms enhance host resistance to pesticides mainly through mechanisms of action, such as degradative metabolism, antioxidant regulation, competitive occupancy, and immunomodulation (33). Insect resistance to pesticides is often accompanied by enhanced antioxidant enzyme activity, as demonstrated by a study showing that carboxylesterases are associated with resistance to chlorpyrifos in brown planthoppers (34). Stimulation of brown planthoppers with chlorpyrifos for eight generations showed an increase in SOD, CAT, and POD in parallel with the increase in LD_50_ values (35). In addition, an association between overexpression of GST-related genes and insecticide resistance has been demonstrated (36). Cytochrome P450, GST, carboxylesterases (CarEs), ABC transporter proteins, cuticular protein encoding genes, and trypsin-related genes have all been reported to be associated with the development of insect resistance to insecticides (37). Previous studies have shown that stimulation of insects with sublethal concentrations of chlorantraniliprole led to effects on development, body weight, metamorphosis, longevity, and fecundity, as well as upregulation of the expression of insect juvenile hormone biosynthesis genes (*JHAMT* and *FPPS*) and downregulation of the expression of the yolk protein gene (*CsVg*) (38). Moreover, stimulation of *Spodoptera frugiperda* with sublethal concentrations of chlorantraniliprole resulted in differences in gene expression among individual larvae, and there was a concentration effect (39). However, in our study, the enhancement of *Spodoptera frugiperda* resistance to beta-cypermethrin by ZG-GX treatment was not associated with physiological adaptations or antioxidant enzyme activities. This suggests that there may be a difference between the mechanism of action of ZG-GX, which leads to enhanced resistance in *Spodoptera frugiperda*, and the enhanced resistance related to antioxidant enzymes in previous studies.

### Amino acid metabolism was significantly changed in ZG-GX-treated *Spodoptera frugiperda*

There is a close correlation and interaction between amino acid metabolism and insect resistance. Amino acids are not only the basic building blocks of protein synthesis in insects but are also associated with insect resistance to pyrethroids. It was shown that tyrosine in the mid-intestine of arthropods has a vital role in resistance to neurotoxic organophosphates, carbamates, and pyrethroids (40). Besides, it has been reported that the metabolism of valine and isoleucine was downregulated in *Spodoptera frugiperda* under ethyl polymyxin stress, which reduced the accumulation of acetyl coenzyme A, a compound involved in the tricarboxylic acid (TCA) cycle. GTP can be produced in the tricarboxylic acid (TCA) cycle to resist ethyl polymyxin stress. Therefore, the metabolism of valine and isoleucine is related to *Spodoptera frugiperda* when resisting ethyl polymyxin stress (6, 41). Arginine phosphorylation with the aid of arginine kinase (AK) affects insect energy utilization—including increased insect demand, altered metabolic pathways, and enhanced antioxidant mechanisms—and consequently influences insect resistance (42). Histidine is one of the most specific substances in insect resistance to pesticides (43). Histidine can be enzymatically catalyzed into histamine, which is widely recognized as an important neurotransmitter. Histamine exerts a variety of physiological functions in the central nervous system, regulating the activity of sodium channels by inducing and inhibiting specific receptors, thereby affecting changes in membrane potential and action potential generation in neurons (44, 45). Histamine can also regulate signal transduction between neurons and participate in the regulation of cell excitability. Meanwhile, beta-cypermethrin, a pyrethroid pesticide, acts on voltage-gated sodium channel proteins in insect neuronal cell membranes (46). It keeps the Na^+^ channels open, allowing Na^+^ to flow into the membrane, which causes a decrease in the domain value potential required for action potentials. This leads to the expression of excitation and uncoordinated movements, which ultimately cause death from insect poisoning (13). Collectively, both histamine and beta-cypermethrin can act on sodium channels. This study revealed that *Stenotrophomonas maltophilia* ZG-GX reduced the number of amino acids in the feces of *Spodoptera frugiperda*. Histidine, most affected by ZG-GX, was reduced to zero μg/g after treatment. The metabolism of histidine includes decarboxylation reactions, acetyl glutamate synthesis, methylation, and amino transfer. Under the action of histidine decarboxylation, histidine is converted into histamine, which, as a neurotransmitter, can act on sodium channels and potentially increase resistance to beta-cypermethrin by changing the opening and closing of sodium channels in *Spodoptera frugiperda*; however, this mechanism requires further validation (Fig. S4).

### Beta-cypermethrin and ZG-GX may enhance *Spodoptera frugiperda* resistance through different pathways

Sykiotis and Bohmann have shown that insect intestinal microbes can enhance antioxidants by influencing the host’s immune system, thereby enhancing the resistance to pesticides (47). Conversely, insecticide stimulation leads to increased insect resistance through genetic mutations, the production of resistance genes, the selection of insects by insecticides, and the inheritance of resistance genes (48). For instance, pyrimethanil and carbendazim mainly enhance the expression of genes related to cellular conjugation (49). Li has previously reported that pesticide stimulation at low concentrations may promote genetic mutations that increase pesticide tolerance in insects (50). In our study, the LD_50_ for beta-cypermethrin was similar between the ZG-GX-treated progeny larvae and beta-cypermethrin-stimulated progeny larvae, but the b-value was smaller in the ZG-GX-treated group. The b-value can reflect the magnitude of insect resistance to pesticides (27), and therefore, the ZG-GX-treated group was more resistant (Table 1). Differences in b-values reflected the different effects of ZG-GX treatment and pesticide stimulation on the pesticide resistance of *Spodoptera frugiperda*.

**Fig 6:**
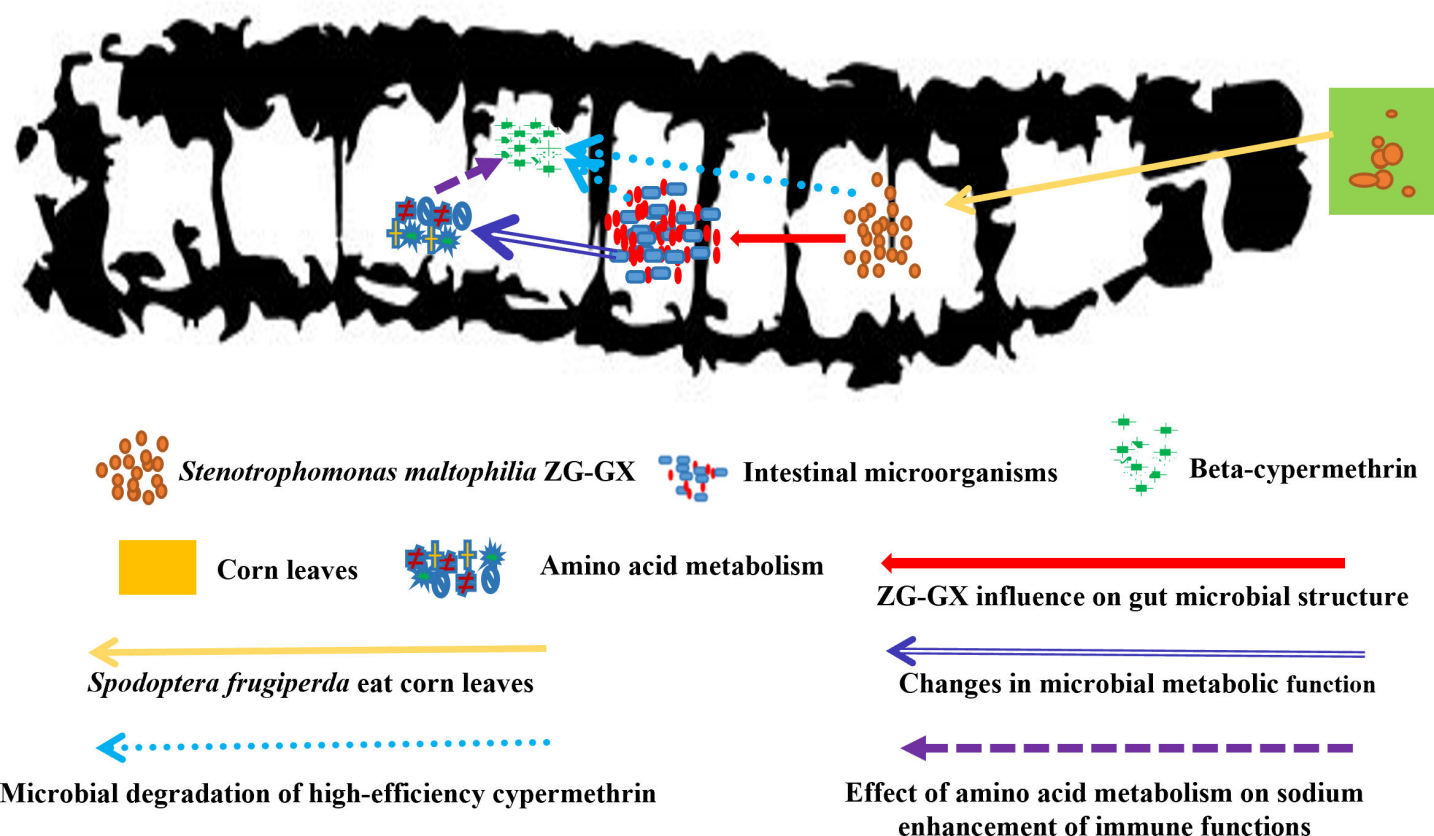
Hypothetical mechanism of S*tenotrophomonas maltophilia* ZG-GX in enhancing the resistance of *Spodoptera frugiperda* to beta-cypermethrin.

Insect resistance to pesticides is often enhanced through alterations in intestinal microbial structure, particularly via a decrease in the species richness of the intestinal microbiota. As Dong pointed out, low concentrations of MD after stimulation led to a reduction in the abundance of core species of the intestinal microbiota of honeybees (51). However, bacteria usually influence the structure of the intestinal microbiota of insects through competitive relationships and metabolic interactions. In our study, the sob after pesticide treatment was decreased. In contrast, the sob in the ZG-GX treatment group was higher than in the control group (*P* < 0.05). It can be concluded that, unlike the effects of low concentrations of pesticides on insect intestinal microorganisms, ZG-GX treatment can enhance their diversity (Fig. 6).

In addition, differences were detected in the amino acid content of *Spodoptera frugiperda* feces after treatment with ZG-GX and co-treatment with pesticide-ZG-GX. All amino acid contents decreased in the feces of *Spodoptera frugiperda* treated with ZG-GX. However, some amino acids, such as isoleucine and glycine, increased in the feces of *Spodoptera frugiperda* co-treated with ZG-GX and beta-cypermethrin. Although the number of amino acids decreased in *Spodoptera frugiperda* feces after co-treatment, ZG-GX caused a greater decrease. Certain pesticides can interfere directly or indirectly with insect amino acid metabolism systems. This interference affects the activity of key enzymes in the amino acid synthesis pathway, resulting in an inadequate supply of amino acids or an imbalance in amino acid metabolism (52).

### Conclusion

In summary, *Stenotrophomonas maltophilia* ZG-GX feeding enhanced the resistance of *Spodoptera frugiperda* to beta-cypermethrin. ZG-GX can reduce the concentration of beta-cypermethrin and alter the structure of the intestinal microbial community and the amino acid metabolism. Meanwhile, there were no significant changes in antioxidant enzyme activities and physiological adaptations of *Spodoptera frugiperda* after feeding with *Stenotrophomonas maltophilia* ZG-GX. Although both *Stenotrophomonas maltophilia* ZG-GX and beta-cypermethrin treatment can enhance the resistance of *Spodoptera frugiperda*, their mechanisms of action were different.

## Data Availability

The data that supports the findings of this study are available in the supporting information of this article. The raw sequence data reported in this paper have been deposited in the Genome Sequence Archive (Genomics, Proteomics & Bioinformatics 2021) in National Genomics Data Center (Nucleic Acids Res 2021), China National Center for Bioinformation / Beijing Institute of Genomics, Chinese Academy of Sciences (GSA: CRA013198 and CRA012814) that are publicly accessible at https://ngdc.cncb.ac.cn/gsa/.

## SUPPLEMENTAL MATERIAL

Supplemental material spectrum.03150-25-s0001.docx Supplemental text, figures, and tables.

ASM does not own the copyrights to Supplemental Material that may be linked to, or accessed through, an article. The authors have granted ASM a non-exclusive, world-wide license to publish the Supplemental Material files. Please contact the corresponding author directly for reuse.
