# Supplementary material for: The intestinal Stenotrophomonas maltophilia ZG-GX enhances the resistance of Spodoptera frugiperda to beta-cypermethrin and decreases the amino acid levels
Source: Microbiol Spectr. 2025 Nov 18;14(1):e03150-25. doi: 10.1128/spectrum.03150-25 (PMC12772350; doi:10.1128/spectrum.03150-25)
Supplement: Supplemental material — Supplemental text, figures, and tables. [file spectrum.03150-25-s0001.docx]

Supplementary Material

The following Supporting Information is available for this article:

***Stenotrophomonas maltophilia* ZG-GX sequencing splicing sequence:**

CCCCCGGTTGGCAAGCGCCCTCCCGAAGGTTAAGCTACCTGCTTCTGGTGCAACAAACTCCCATGGTGTGACGGGCGGTGTGTACAAGGCCCGGGAACGTATTCACCGCAGCAATGCTGATCTGCGATTACTAGCGATTCCGACTTCATGGAGTCGAGTTGCAGACTCCAATCCGGACTGAGATAGGGTTTCTGGGATTGGCTTACCGTCGCCGGCTTGCAGCCCTCTGTCCCTACCATTGTAGTACGTGTGTAGCCCTGGCCGTAAGGGCCATGATGACTTGACGTCATCCCCACCTTCCTCCGGTTTGTCACCGGCGGTCTCCTTAAAGTTCCCACCATTACGTGCTGGCAACTAAGGACAAGGGTTGCGCTCGTTGCGGGACTTAACCCAACATCTCACGACACGAGCTGACGACAGCCATGCAGCACCTGTGTTCGAGTTCCCGAAGGCACCCATCCATCTCTGGAAAGTTCTCGACATGTCAAGGCCAGGTAAGGTTCTTCCCGTTGCATCGAATTAAACCACATACTCCACCGCTTGTGCGGGCCCCCGTCAATTCCTTTGAGTTTCAGTCTTGCGACCGTACTCCCCAGGCGGCGAACTTAACGCGTTAGCTTCGATACTGCGTGCCAAATTGCACCCAACATCCAGTTCGCATCGTTTAGGGCGTGGACTACCAGGGTATCTAATCCTGTTTGCTCCCCACGCTTTCGTGCCTCAGTGTCAGTGTTGGTCCAGGTAGCTGCCTTCGCCATGGATGTTCCTCCTGATCTCTACGCATTTCACTGCTACACCAGGAATTCCGCTACCCTCTACCACACTCTAGTCGCCCAGTATCCACTGCAGTTCCCAGGTTGAGCCCAGGGCTTTCACAACGGACTTAAACGACCACCTACGCACGCTTTACGCCCAGTAATTCCGAGTAACGCTTGCACCCTTCGTATTACCGCGGCTGCTGGCACGAAGTTAGCCGGTGCTTATTCTTTGGGTACCGTCATCCCAACCGGGTATTAGCCAGCTGGATTTCTTTCCCAACAAAAGGGCTTTACAACCCGAAGGCCTTCTTCACCCACGCGGTATGGCTGGATCAGGCTTGCGCCCATTGTCCAATATTCCCCACTGCTGCCTCCCGTAGGAGTCTGGACCGTGTCTCAGTTCCAGTGTGGCTGATCATCCTCTCAGACCAGCTACGGATCGTCGCCTTGGTGGGCCTTTACCCCGCCAACTAGCTAATCCGACATCGGCTCATTCAATCGCGCAAGGTCCGAAGATCCCCTGCTTTCACCCGTAGGTCGTATGCGGTATTAGCGTAAGTTTCCCTACGTTATCCCCCACGACAGAGTAGATTCCGATGTATTCCTCACCCGTCCGCCACTCGCCACCCAGGGAGCAAGCTCTTCTGTGCTGCCGTTCGACTTGCATGTGTAGCCTACCGCATTCCC

Sequence comparison of *Stenotrophomonas maltophilia* ZG-GX with the CDS sequence of *Stenotrophomonas maltophilia PVC-SHT* results in 99.58% similarity.

*Stenotrophomonas maltophilia PVC-SHT:*

GenBank:MK300721.1

[GenBank](https://www.ncbi.nlm.nih.gov/nuccore/HM991259.1?report=genbank) [Graphics](https://www.ncbi.nlm.nih.gov/nuccore/HM991259.1?report=graph)

>MK300721.1 *Stenotrophomonas maltophilia strain PVC-SHT* 16S ribosomal RNA gene, partial sequence


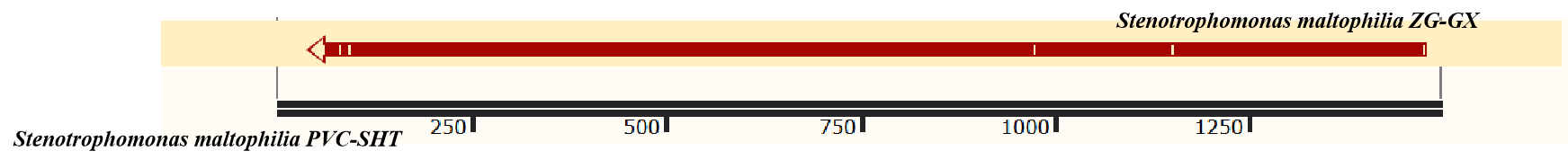


**Supplementary Table 1.** Data preprocessing statistics and quality control of *Spodoptera frugiperda* gut microbial sequencing results

| **Sample Name** | **Raw Reads** | **Clean Reads** | **Raw Tags** | **Clean Tags** | **Chimera** | **Effective Tags** | **Effective Ratio (%)** |
| --- | --- | --- | --- | --- | --- | --- | --- |
| **CK1-1** | 112000 | 111972 | 111781 | 111454 | 11501 | 99953 | 89.24 |
| **CK1-2** | 119506 | 119485 | 119234 | 118806 | 10378 | 108428 | 90.73 |
| **CK1-3** | 121026 | 121005 | 120763 | 120375 | 10305 | 110070 | 90.95 |
| **CK2-1** | 123074 | 123055 | 122781 | 122434 | 11379 | 111055 | 90.23 |
| **CK2-2** | 117340 | 117317 | 117072 | 116675 | 11452 | 105223 | 89.67 |
| **CK2-3** | 123074 | 123032 | 122788 | 122254 | 11654 | 110600 | 89.86 |
| **Pf1-1** | 93133 | 93117 | 92954 | 92576 | 8455 | 84121 | 90.32 |
| **Pf1-2** | 121524 | 121505 | 121259 | 120791 | 13248 | 107543 | 88.5 |
| **Pf1-3** | 123074 | 123052 | 122789 | 122330 | 11973 | 110357 | 89.67 |
| **Pf2-1** | 114550 | 114536 | 114313 | 113935 | 10656 | 103279 | 90.16 |
| **Pf2-2** | 114621 | 114597 | 114407 | 114203 | 10805 | 103398 | 90.21 |
| **Pf2-3** | 123074 | 123057 | 122845 | 122502 | 11658 | 110844 | 90.06 |
| **Pf3-1** | 123074 | 123054 | 122844 | 122519 | 11180 | 111339 | 90.47 |
| **Pf3-2** | 123074 | 123055 | 122836 | 122508 | 13155 | 109353 | 88.85 |
| **Pf3-3** | 121828 | 121815 | 121583 | 121207 | 11130 | 110077 | 90.35 |
| **S1-1** | 123074 | 123050 | 122780 | 122356 | 12884 | 109472 | 88.95 |
| **S1-2** | 123074 | 123055 | 122821 | 122488 | 11329 | 111159 | 90.32 |
| **S1-3** | 123074 | 123050 | 122782 | 122462 | 12155 | 110307 | 89.63 |
| **S2-1** | 116295 | 116281 | 116034 | 115678 | 11042 | 104636 | 89.97 |
| **S2-2** | 117321 | 117299 | 117063 | 116738 | 12334 | 104404 | 88.99 |
| **S2-3** | 117888 | 117863 | 117648 | 117380 | 11372 | 106008 | 89.92 |
| **F1** | 113640 | 113640 | 112835 | 112508 | 2645 | 109863 | 96.68 |
| **F2** | 113836 | 113836 | 113113 | 112708 | 5061 | 107647 | 94.56 |
| **F3** | 104535 | 104535 | 103845 | 103157 | 4194 | 98963 | 94.67 |

Raw PE: Raw Log of Pair-end Reads, Clean PE: Logarithm of high-quality Pair-end Reads obtained after QC filtering, Raw Tags: The number of raw Tags obtained after overlap assembly, Clean Tags: Number of high quality Tags obtained after Tags quality control, Chimera: Number of chimeric Tags detected during the OTU clustering process, Effective Tags: Number of high-quality Tags after removal of chimeras, i.e., valid Tags for subsequent analyses,Effective Ratio (%): Number of High Quality Tags as a Percentage of Raw PE Reads.

**Supplementary Table 2.** The top 30 bacterial orders in terms of abundance in the 16SRNA sequencing results of *Spodoptera frugiperda* gut microorganisms and the associated abundance share values (%).

|  | **CK1** | **CK2** | **Pf1** | **Pf2** | **Pf3** | **S1** | **S2** |
| --- | --- | --- | --- | --- | --- | --- | --- |
| ***Aeromonadales*** | 0.6593 | 0.6632 | 0.4194 | 0.6168 | 0.6075 | 0.5603 | 0.0057 |
| ***Bacillales*** | 17.6625 | 18.4463 | 10.8851 | 15.4733 | 19.7364 | 17.3120 | 0.2947 |
| ***Bacteroidales*** | 12.2175 | 10.7745 | 7.4854 | 10.2148 | 10.6600 | 8.9693 | 3.1798 |
| ***Betaproteobacteriales*** | 5.3723 | 4.8215 | 3.7475 | 5.2585 | 5.7498 | 5.5986 | 2.9059 |
| ***Campylobacterales*** | 0.4874 | 0.4992 | 0.2744 | 0.4499 | 0.4191 | 0.4128 | 0.0004 |
| ***Caulobacterales*** | 1.4610 | 1.7250 | 1.1732 | 1.4738 | 1.5225 | 1.3911 | 0.5126 |
| ***Chitinophagales*** | 1.7424 | 2.2690 | 1.5091 | 1.9057 | 1.9291 | 1.8435 | 0.0012 |
| ***Chloroplast*** | 4.5613 | 4.5318 | 1.6428 | 3.7905 | 3.8922 | 2.2843 | 1.9341 |
| ***Clostridiales*** | 8.1467 | 8.2398 | 5.1758 | 8.0798 | 8.2902 | 7.6794 | 5.7947 |
| ***Coriobacteria*** | 0.5686 | 0.6561 | 0.3482 | 0.4745 | 0.5882 | 0.7468 | 0.1291 |
| ***Corynebacteriales*** | 0.2442 | 0.4820 | 0.3486 | 0.3238 | 0.4191 | 0.3730 | 0.0728 |
| ***Desulfovibrionales*** | 0.3948 | 0.3927 | 0.2701 | 0.2580 | 0.4023 | 0.3766 | 0.0255 |
| ***Enterobacteriales*** | 1.3568 | 3.1334 | 4.6818 | 2.2872 | 2.9325 | 7.5099 | 2.6015 |
| ***Erysipelotrichales*** | 5.6360 | 6.0904 | 3.4099 | 4.8815 | 6.0603 | 5.3310 | 0.1444 |
| ***Flavobacteriales*** | 0.8539 | 0.9454 | 0.8426 | 1.0773 | 1.1179 | 1.5550 | 2.6892 |
| ***Fusobacteriales*** | 2.6101 | 0.7849 | 0.4588 | 0.6304 | 0.8671 | 0.7120 | 0.0043 |
| ***Lactobacillales*** | 7.1440 | 9.5093 | 38.4041 | 17.2833 | 4.4114 | 6.6702 | 42.9120 |
| ***Micrococcales*** | 1.1959 | 0.5107 | 0.3338 | 0.5018 | 0.5360 | 0.5687 | 0.0657 |
| ***Pasteurellales*** | 1.8279 | 0.5829 | 0.3181 | 0.5104 | 0.7279 | 0.5988 | 0.0392 |
| ***Pseudomonadales*** | 1.9915 | 2.0927 | 1.6655 | 1.6246 | 3.5347 | 4.1418 | 16.1687 |
| ***Rhizobiales*** | 1.6240 | 1.8266 | 1.8071 | 1.6971 | 2.2914 | 1.9715 | 0.5991 |
| ***Rhodobacterales*** | 0.4418 | 0.5011 | 0.3853 | 0.3999 | 0.4411 | 0.3713 | 0.6567 |
| ***Rickettsiales*** | 1.2242 | 1.3999 | 0.9610 | 1.3331 | 1.4828 | 1.1792 | 0.1915 |
| ***Saccharimonadales*** | 0.5834 | 1.7134 | 1.8197 | 2.8434 | 3.6310 | 3.4931 | 0.0136 |
| ***Selenomonadales*** | 1.8332 | 0.6249 | 0.4219 | 0.4629 | 0.4742 | 0.5046 | 0.9271 |
| ***Sphingobacteriales*** | 0.1551 | 0.7222 | 0.5784 | 0.3316 | 0.3587 | 1.3127 | 1.2438 |
| ***Sphingomonadales*** | 0.2752 | 0.3876 | 0.3208 | 0.4278 | 0.4814 | 0.3769 | 1.0290 |
| ***Verrucomicro*** | 0.6066 | 0.6559 | 0.5714 | 0.7271 | 0.6556 | 0.5435 | 0.0003 |
| ***Vibrionales*** | 5.3135 | 6.0395 | 3.8852 | 6.3542 | 6.0915 | 5.4501 | 13.8001 |
| ***Xanthomona*** | 0.2524 | 0.3962 | 0.1652 | 0.2997 | 0.3326 | 0.8369 | 1.1996 |

**Supplementary Table 3.** The top 60 bacterial families in terms of abundance in the 16SRNA sequencing results of *Spodoptera frugiperda* gut microorganisms and the associated abundance share values (%).

|  | **CK1** | **CK2** | **Pf1** | **Pf2** | **Pf3** | **S1** | **S2** |
| --- | --- | --- | --- | --- | --- | --- | --- |
| ***Acidaminococcaceae*** | 0.2667 | 0.2717 | 0.3125 | 0.1450 | 0.2385 | 0.1921 | 0.2487 |
| ***Actinomycetaceae*** | 0.5816 | 0.9110 | 0.0883 | 0.0120 | 0.0269 | 0.0375 | 0.0750 |
| ***Aeromonadaceae*** | 0.3726 | 0.3144 | 0.3341 | 0.1984 | 0.3162 | 0.3445 | 0.2974 |
| ***Akkermansiaceae*** | 0.5148 | 0.5705 | 0.5616 | 0.4806 | 0.6053 | 0.5026 | 0.4708 |
| ***Anaerolineaceae*** | 0.1711 | 0.1714 | 0.2129 | 0.0567 | 0.1196 | 0.1668 | 0.1152 |
| ***Atopobiaceae*** | 0.4978 | 0.4974 | 0.5307 | 0.2479 | 0.3771 | 0.4595 | 0.5563 |
| ***Bacillaceae*** | 0.1726 | 0.0892 | 0.1174 | 0.0798 | 0.1145 | 0.1549 | 0.2168 |
| ***Bacteroidaceae*** | 1.3373 | 1.0011 | 0.9331 | 0.7753 | 1.1471 | 1.0469 | 1.0911 |
| ***Beijerinckiaceae*** | 0.7013 | 0.5298 | 0.6659 | 0.4001 | 0.6099 | 0.6034 | 0.4518 |
| ***Bifidobacteriaceae*** | 0.3230 | 0.3175 | 0.3504 | 0.1608 | 0.2486 | 0.3450 | 0.2074 |
| ***Burkholderiaceae*** | 2.1495 | 1.7705 | 3.3920 | 2.7179 | 3.9642 | 4.1325 | 4.3285 |
| ***Caulobacteraceae*** | 1.4610 | 1.2665 | 1.7250 | 1.1690 | 1.4667 | 1.5127 | 1.3907 |
| ***Chitinophagaceae*** | 1.6728 | 1.7324 | 2.1501 | 1.4370 | 1.7872 | 1.8647 | 1.7613 |
| ***Christensenellaceae*** | 0.1429 | 0.1522 | 0.1723 | 0.0797 | 0.2450 | 0.1880 | 0.1783 |
| ***Chthoniobacteraceae*** | 0.2952 | 0.1492 | 0.2782 | 0.1675 | 0.1953 | 0.2582 | 0.2641 |
| ***Clostridiaceae_1*** | 0.1008 | 0.0580 | 0.2127 | 0.2114 | 0.5284 | 0.3714 | 0.2556 |
| ***Clostridiales_vadinBB60_group*** | 0.3215 | 0.2336 | 0.2096 | 0.1540 | 0.2276 | 0.2128 | 0.1694 |
| ***Corynebacteriaceae*** | 0.2279 | 0.3950 | 0.4496 | 0.3170 | 0.2858 | 0.3261 | 0.3374 |
| ***Desulfovibrionaceae*** | 0.3948 | 0.3349 | 0.3927 | 0.2701 | 0.2538 | 0.4023 | 0.3766 |
| ***Enterobacteriaceae*** | 1.3568 | 1.2423 | 3.1334 | 4.6818 | 2.2872 | 2.9325 | 7.5099 |
| ***Enterococcaceae*** | 2.1865 | 5.9115 | 7.8380 | 37.4729 | 15.9726 | 2.8119 | 5.1417 |
| ***Erysipelotrichaceae*** | 5.6360 | 5.5804 | 6.0904 | 3.4099 | 4.8815 | 6.0603 | 5.3310 |
| ***Family_XI*** | 0.4825 | 0.5499 | 0.6037 | 0.5081 | 0.4930 | 0.4990 | 0.5280 |
| ***Flavobacteriaceae*** | 0.2595 | 0.3045 | 0.2566 | 0.2899 | 0.4462 | 0.2300 | 0.4915 |
| ***Fusobacteriaceae*** | 1.0957 | 1.0977 | 0.7770 | 0.4531 | 0.6286 | 0.8492 | 0.6803 |
| ***Lachnospiraceae*** | 3.7739 | 3.1351 | 3.7616 | 2.1769 | 3.1280 | 3.6923 | 3.3497 |
| ***Lactobacillaceae*** | 0.3333 | 0.3026 | 0.4569 | 0.1854 | 0.3844 | 0.3722 | 0.3567 |
| ***Leptotrichiaceae*** | 1.5145 | 1.5707 | 0.0079 | 0.0057 | 0.0018 | 0.0180 | 0.0305 |
| ***Micrococcaceae*** | 1.0559 | 0.8244 | 0.3601 | 0.2675 | 0.3848 | 0.4195 | 0.3791 |
| ***Mitochondria*** | 1.2242 | 1.4522 | 1.3999 | 0.9569 | 1.3256 | 1.4788 | 1.1792 |
| ***Moraxellaceae*** | 1.1950 | 0.7400 | 0.9359 | 0.9574 | 0.8167 | 2.4335 | 2.5870 |
| ***Muribaculaceae*** | 1.3196 | 1.2360 | 1.6657 | 0.8044 | 1.4313 | 1.3071 | 1.0105 |
| ***Neisseriaceae*** | 2.9894 | 3.8003 | 1.3129 | 0.9437 | 1.2077 | 1.5080 | 1.1470 |
| ***NS9_marine_group*** | 0.1542 | 0.1535 | 0.2309 | 0.1169 | 0.1522 | 0.1687 | 0.1894 |
| ***Pasteurellaceae*** | 1.8279 | 1.9951 | 0.5829 | 0.3181 | 0.5104 | 0.7279 | 0.5988 |
| ***Peptostreptococcaceae*** | 0.4353 | 0.5022 | 0.5252 | 0.2495 | 0.4355 | 0.4830 | 0.5321 |
| ***Pirellulaceae*** | 0.1755 | 0.1517 | 0.1361 | 0.1667 | 0.1255 | 0.1001 | 0.1418 |
| ***Planococcaceae*** | 0.2058 | 0.1848 | 0.1318 | 0.1072 | 0.5465 | 0.1652 | 0.4754 |
| ***Porphyromonadaceae*** | 0.2871 | 0.8682 | 0.0604 | 0.0105 | 0.0081 | 0.0250 | 0.0041 |
| ***Prevotellaceae*** | 7.5735 | 10.1253 | 6.3227 | 4.4753 | 5.9286 | 6.6626 | 5.2926 |
| ***Pseudomonadaceae*** | 0.7964 | 0.9016 | 1.1567 | 0.7081 | 0.8079 | 1.1012 | 1.5548 |
| ***Rhizobiaceae*** | 0.4572 | 0.4462 | 0.6692 | 1.0555 | 0.7064 | 1.1159 | 1.0474 |
| ***Rhodobacteraceae*** | 0.4418 | 0.3691 | 0.5011 | 0.3853 | 0.3999 | 0.4411 | 0.3713 |
| ***Rikenellaceae*** | 0.7164 | 0.7386 | 0.8255 | 0.6363 | 0.8501 | 0.7089 | 0.7501 |
| ***Ruminococcaceae*** | 2.7935 | 2.4038 | 2.6032 | 1.5691 | 2.7245 | 2.5940 | 2.4436 |
| ***Saccharimonadaceae*** | 0.4754 | 0.5678 | 1.4417 | 1.6066 | 2.6281 | 3.2999 | 3.1445 |
| ***Solibacteraceae_Subgroup_3*** | 0.3686 | 0.1193 | 0.2172 | 0.1650 | 0.1778 | 0.2097 | 0.2386 |
| ***Sphingobacteriaceae*** | 0.1260 | 0.0613 | 0.6737 | 0.5404 | 0.2897 | 0.2849 | 1.2932 |
| ***Sphingomonadaceae*** | 0.2752 | 0.2856 | 0.3876 | 0.3208 | 0.4278 | 0.4814 | 0.3769 |
| ***Staphylococcaceae*** | 17.1058 | 15.8151 | 18.0769 | 10.2748 | 14.6829 | 19.2966 | 16.4111 |
| ***Streptococcaceae*** | 3.6987 | 3.6096 | 1.0689 | 0.6825 | 0.8408 | 1.0845 | 1.0508 |
| ***Succinivibrionaceae*** | 0.2867 | 0.3509 | 0.3291 | 0.2210 | 0.3006 | 0.2630 | 0.2630 |
| ***Sulfurovaceae*** | 0.2092 | 0.2461 | 0.2625 | 0.1474 | 0.2197 | 0.1974 | 0.1964 |
| ***Tannerellaceae*** | 0.2399 | 0.3004 | 0.3154 | 0.2572 | 0.2699 | 0.3086 | 0.2845 |
| ***Veillonellaceae*** | 1.5665 | 2.5265 | 0.3124 | 0.2769 | 0.2244 | 0.2821 | 0.2559 |
| ***Vibrionaceae*** | 5.3135 | 4.9740 | 6.0395 | 3.8852 | 6.3542 | 6.0915 | 5.4501 |
| ***WD2101_soil_group*** | 0.4986 | 0.1842 | 0.3241 | 0.2043 | 0.3158 | 0.3549 | 0.2749 |
| ***Weeksellaceae*** | 0.4345 | 0.4364 | 0.4557 | 0.4301 | 0.4564 | 0.7174 | 0.8693 |
| ***Xanthobacteraceae*** | 0.3253 | 0.2847 | 0.3198 | 0.2472 | 0.2561 | 0.3163 | 0.2289 |
| ***Xanthomonadaceae*** | 0.1318 | 0.0971 | 0.3136 | 0.1302 | 0.2203 | 0.1957 | 0.7214 |

**Supplementary Table 4.** The top 60 bacterial genera in terms of abundance in the 16SRNA sequencing results of *Spodoptera frugiperda* gut microorganisms and the associated abundance share values (%).

|  | **CK1** | **CK2** | **Pf1** | **Pf2** | **Pf3** | **S1** | **S2** |
| --- | --- | --- | --- | --- | --- | --- | --- |
| ***Acetitomaculum*** | 0.5639 | 0.4825 | 0.6231 | 0.3175 | 0.4613 | 0.5736 | 0.4929 |
| ***Acinetobacter*** | 1.0655 | 0.5947 | 0.8503 | 0.8854 | 0.7077 | 2.2766 | 2.4423 |
| ***Actinomyces*** | 0.5519 | 0.8679 | 0.0098 | 0.0044 | 0.0028 | 0.0119 | 0.0081 |
| ***Aeromonas*** | 0.3726 | 0.3066 | 0.3229 | 0.1941 | 0.3059 | 0.3427 | 0.2974 |
| ***Agathobacter*** | 0.1536 | 0.1583 | 0.1670 | 0.0955 | 0.1574 | 0.1393 | 0.1101 |
| ***Akkermansia*** | 0.5148 | 0.5705 | 0.5616 | 0.4806 | 0.6053 | 0.5026 | 0.4708 |
| ***Alistipes*** | 0.4362 | 0.4152 | 0.5078 | 0.3929 | 0.4710 | 0.4059 | 0.4647 |
| ***Alloprevotella*** | 0.1991 | 0.2674 | 0.2000 | 0.1363 | 0.3143 | 0.1700 | 0.1533 |
| ***Allorhizobium-Neorhizobium-Pararhizobium-Rhizobium*** | 0.0870 | 0.1331 | 0.2506 | 0.6524 | 0.2970 | 0.3278 | 0.3717 |
| ***Bacteroides*** | 1.3373 | 1.0011 | 0.9331 | 0.7753 | 1.1471 | 1.0469 | 1.0911 |
| ***Bergeyella*** | 0.2980 | 0.2781 | 0.2784 | 0.2533 | 0.3129 | 0.2584 | 0.2543 |
| ***Bifidobacterium*** | 0.3068 | 0.2671 | 0.2976 | 0.1468 | 0.2349 | 0.3174 | 0.2036 |
| ***Blautia*** | 0.5196 | 0.2441 | 0.3956 | 0.2363 | 0.2764 | 0.3588 | 0.3555 |
| ***Bradyrhizobium*** | 0.2211 | 0.2152 | 0.2668 | 0.1830 | 0.2003 | 0.2611 | 0.1839 |
| ***Burkholderia-Caballeronia-Paraburkholderia*** | 1.3464 | 1.0353 | 1.1694 | 0.7288 | 1.0107 | 1.4071 | 1.2401 |
| ***Candidatus_Saccharimonas*** | 0.3749 | 0.3842 | 0.4415 | 0.3250 | 0.2922 | 0.3277 | 0.3908 |
| ***Cetobacterium*** | 0.8054 | 0.6591 | 0.7159 | 0.4145 | 0.5500 | 0.7686 | 0.6182 |
| ***Chryseobacterium*** | 0.0695 | 0.0898 | 0.1121 | 0.0640 | 0.0698 | 0.3907 | 0.4488 |
| ***Comamonas*** | 0.0297 | 0.0252 | 0.9766 | 0.4010 | 1.2123 | 0.7132 | 0.8739 |
| ***Corynebacterium_1*** | 0.1425 | 0.2120 | 0.3096 | 0.1565 | 0.2160 | 0.2138 | 0.1820 |
| ***Enhydrobacter*** | 0.0432 | 0.0659 | 0.0309 | 0.0350 | 0.0460 | 0.0809 | 0.0621 |
| ***Enterobacter*** | 0.4180 | 0.3721 | 2.1871 | 3.5300 | 1.4068 | 1.9508 | 6.5948 |
| ***Enterococcus*** | 2.1861 | 5.9115 | 7.8245 | 37.4640 | 15.9680 | 2.8105 | 5.1349 |
| ***Erysipelotrichaceae_UCG-002*** | 3.9185 | 3.9377 | 4.3504 | 2.2986 | 3.4212 | 4.3767 | 3.6983 |
| ***Erysipelotrichaceae_UCG-009*** | 0.2530 | 0.3904 | 0.3675 | 0.1548 | 0.2543 | 0.3607 | 0.2786 |
| ***Escherichia-Shigella*** | 0.3302 | 0.1397 | 0.1612 | 0.1133 | 0.1384 | 0.1539 | 0.1394 |
| ***Faecalibacterium*** | 0.2465 | 0.0927 | 0.1289 | 0.0754 | 0.1243 | 0.1058 | 0.0724 |
| ***Faecalibaculum*** | 0.3583 | 0.3584 | 0.3587 | 0.2019 | 0.2416 | 0.4166 | 0.3217 |
| ***Haemophilus*** | 1.2153 | 1.5363 | 0.0029 | 0.0036 | 0.0042 | 0.0125 | 0.0182 |
| ***Klebsiella*** | 0.1247 | 0.1760 | 0.1734 | 0.4806 | 0.1773 | 0.1774 | 0.1538 |
| ***Lachnospiraceae_NK3A20_group*** | 0.7204 | 0.6081 | 0.7523 | 0.4347 | 0.6945 | 0.7334 | 0.6910 |
| ***Lactobacillus*** | 0.3054 | 0.2936 | 0.4277 | 0.1693 | 0.3297 | 0.3537 | 0.3362 |
| ***Leptotrichia*** | 1.5145 | 1.5707 | 0.0079 | 0.0057 | 0.0018 | 0.0180 | 0.0305 |
| ***Methylobacterium*** | 0.5335 | 0.4448 | 0.5639 | 0.3117 | 0.5571 | 0.4970 | 0.3465 |
| ***Neisseria*** | 2.9791 | 3.7129 | 1.3031 | 0.9353 | 1.2031 | 1.5073 | 1.1470 |
| ***Olsenella*** | 0.3902 | 0.3367 | 0.4403 | 0.1894 | 0.3179 | 0.4002 | 0.4612 |
| ***Other*** | 13.6706 | 12.3159 | 11.9613 | 9.4155 | 12.6958 | 12.8983 | 13.2903 |
|  |  |  |  |  |  |  |  |
| ***Parabacteroides*** | 0.2210 | 0.2582 | 0.2958 | 0.2344 | 0.2401 | 0.2649 | 0.2304 |
| ***Photobacterium*** | 4.5653 | 4.2248 | 5.2275 | 3.2888 | 4.8462 | 5.1841 | 4.7372 |
| ***Prevotella*** | 0.2289 | 0.6067 | 0.2481 | 0.2208 | 0.1676 | 0.1861 | 0.1845 |
| ***Prevotella_1*** | 2.1849 | 1.9865 | 1.7373 | 1.2394 | 1.7210 | 2.0619 | 1.6787 |
| ***Prevotella_7*** | 3.3649 | 6.1553 | 2.8349 | 2.0062 | 2.5281 | 3.0036 | 2.2948 |
| ***Prevotella_9*** | 0.2403 | 0.2027 | 0.2432 | 0.1566 | 0.1897 | 0.2219 | 0.2307 |
| ***Prevotellaceae_YAB2003_group*** | 0.2229 | 0.2674 | 0.3204 | 0.2273 | 0.3739 | 0.2932 | 0.2791 |
| ***Pseudomonas*** | 0.7964 | 0.9016 | 1.1560 | 0.7081 | 0.8079 | 1.1012 | 1.5548 |
| ***Ralstonia*** | 0.2305 | 0.2490 | 0.5894 | 0.7575 | 0.9624 | 0.9610 | 0.7536 |
| ***Rikenellaceae_RC9_gut_group*** | 0.2429 | 0.3025 | 0.3077 | 0.2221 | 0.3621 | 0.2687 | 0.2518 |
| ***Rodentibacter*** | 0.4375 | 0.3090 | 0.4248 | 0.2334 | 0.3975 | 0.5237 | 0.4250 |
| ***Romboutsia*** | 0.3158 | 0.3389 | 0.4017 | 0.1969 | 0.3314 | 0.3810 | 0.4374 |
| ***Ruminococcaceae_UCG-005*** | 0.4221 | 0.3951 | 0.5288 | 0.3105 | 0.5739 | 0.5263 | 0.4806 |
| ***Ruminococcaceae_UCG-014*** | 0.7599 | 0.8265 | 0.7019 | 0.4945 | 0.6232 | 0.7062 | 0.7281 |
| ***Sediminibacterium*** | 1.3132 | 1.5692 | 1.8341 | 1.2425 | 1.4387 | 1.6675 | 1.4596 |
| ***Sphingobacterium*** | 0.0583 | 0.0292 | 0.1683 | 0.3530 | 0.1722 | 0.1940 | 1.1153 |
| ***Sphingomonas*** | 0.2040 | 0.1699 | 0.2617 | 0.2045 | 0.2584 | 0.3794 | 0.2718 |
| ***Staphylococcus*** | 17.0163 | 15.7671 | 18.0018 | 10.2432 | 14.6251 | 19.2061 | 16.3418 |
| ***Stenotrophomonas*** | 0.0870 | 0.0712 | 0.2738 | 0.0901 | 0.1561 | 0.1302 | 0.6551 |
| ***Streptococcus*** | 3.5551 | 3.4923 | 0.9555 | 0.5996 | 0.7334 | 0.9683 | 0.9374 |
| ***Succinivibrio*** | 0.2460 | 0.3105 | 0.3144 | 0.2175 | 0.2802 | 0.2522 | 0.2420 |
| ***Sulfurovum*** | 0.2092 | 0.2461 | 0.2625 | 0.1474 | 0.2197 | 0.1974 | 0.1964 |
| ***Unclassified*** | 22.5126 | 17.9551 | 21.6723 | 13.3819 | 20.6883 | 22.7862 | 20.3790 |
| ***Veillonella*** | 1.2548 | 2.1808 | 0.0290 | 0.0147 | 0.0171 | 0.0231 | 0.0192 |
| ***Vibrio*** | 0.7077 | 0.7139 | 0.7782 | 0.5463 | 0.8615 | 0.8452 | 0.6842 |


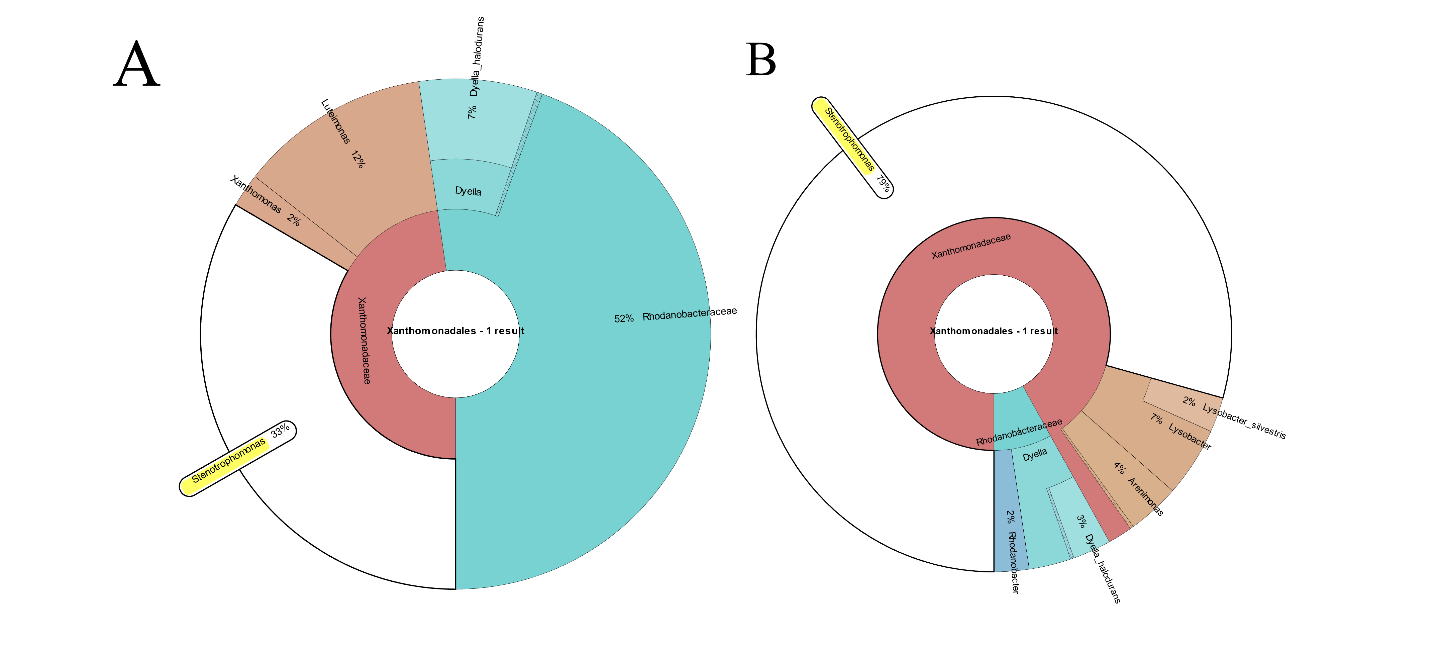


**Fig. S1：A:** Krona pie chart of *Mesorhizobium* species in the control group (CK1); B: Krona pie chart of *Mesorhizobium* species in the pesticide-treated group (Pf1).


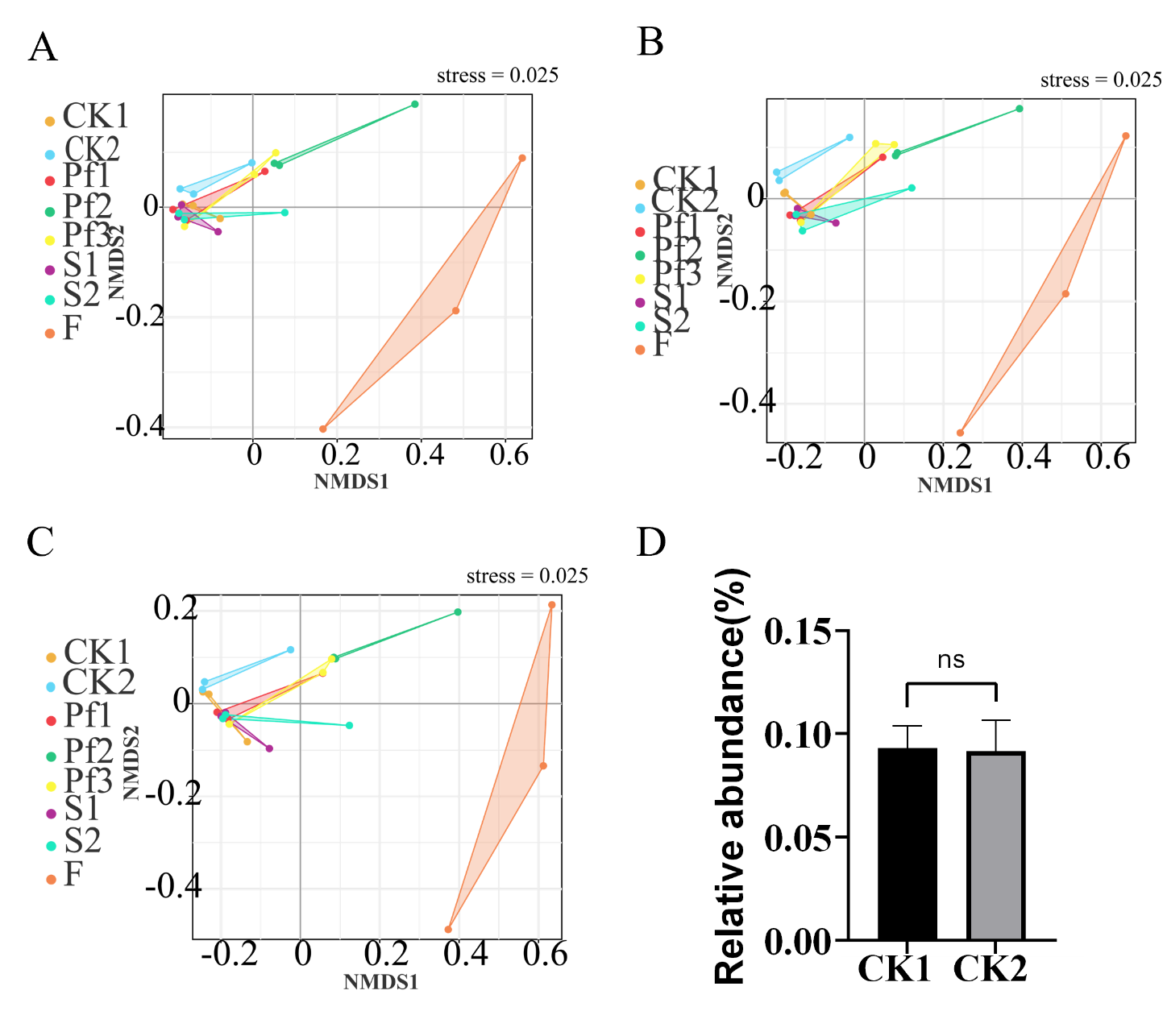


**Fig. S2.** Effects of ZG-GX and beta-cypermethrin on the gut microbial structure of *Spodoptera* *frugiperda*, including overall changes in gut microbial structure versus changes at the level of order, family, and genus. (A-C) NMDS clustering of gut microbial order, family and genus level richness (with group F). (D) Abundance percentage (%) of *Stenotrophomonas maltophilia* spp. in the CK1 and CK2 groups of grass greedy gut sequencing results.


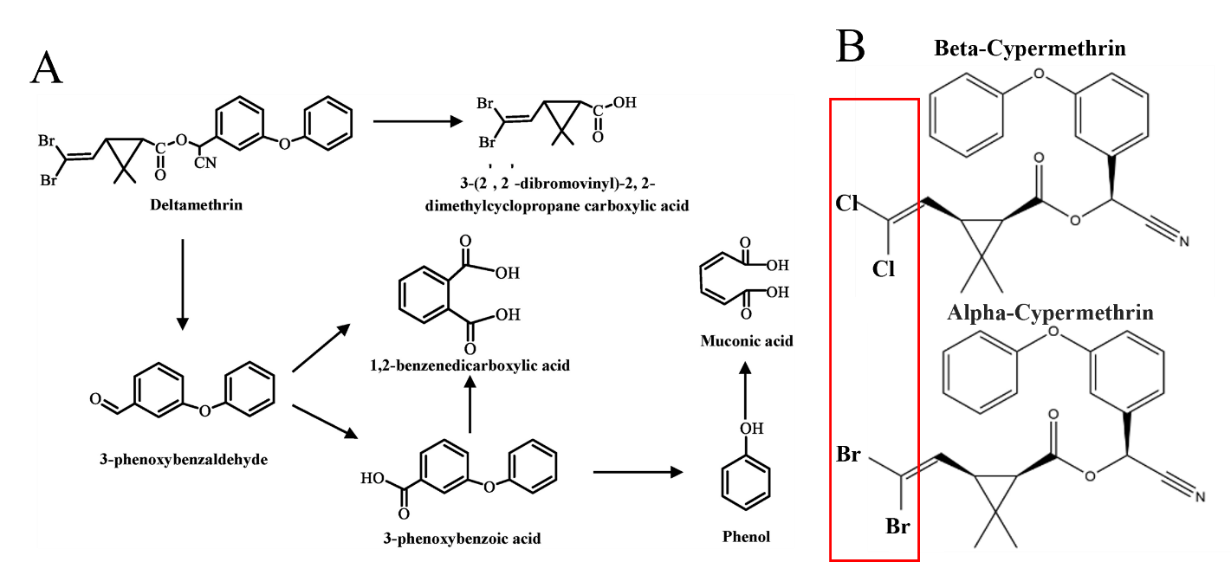


**Fig. S3.** Degradation process of pyrethroids by *Stenotrophomonas* *maltophilia* and molecular structure of pyrethroids: (A) Degradation of deltamethrin by the *Stenotrophomonas maltophilia* XQ08 (Wu et al., 2009); (B) The molecular structures of deltamethrin and beta-cypermethrin have a very high degree of similarity.

**
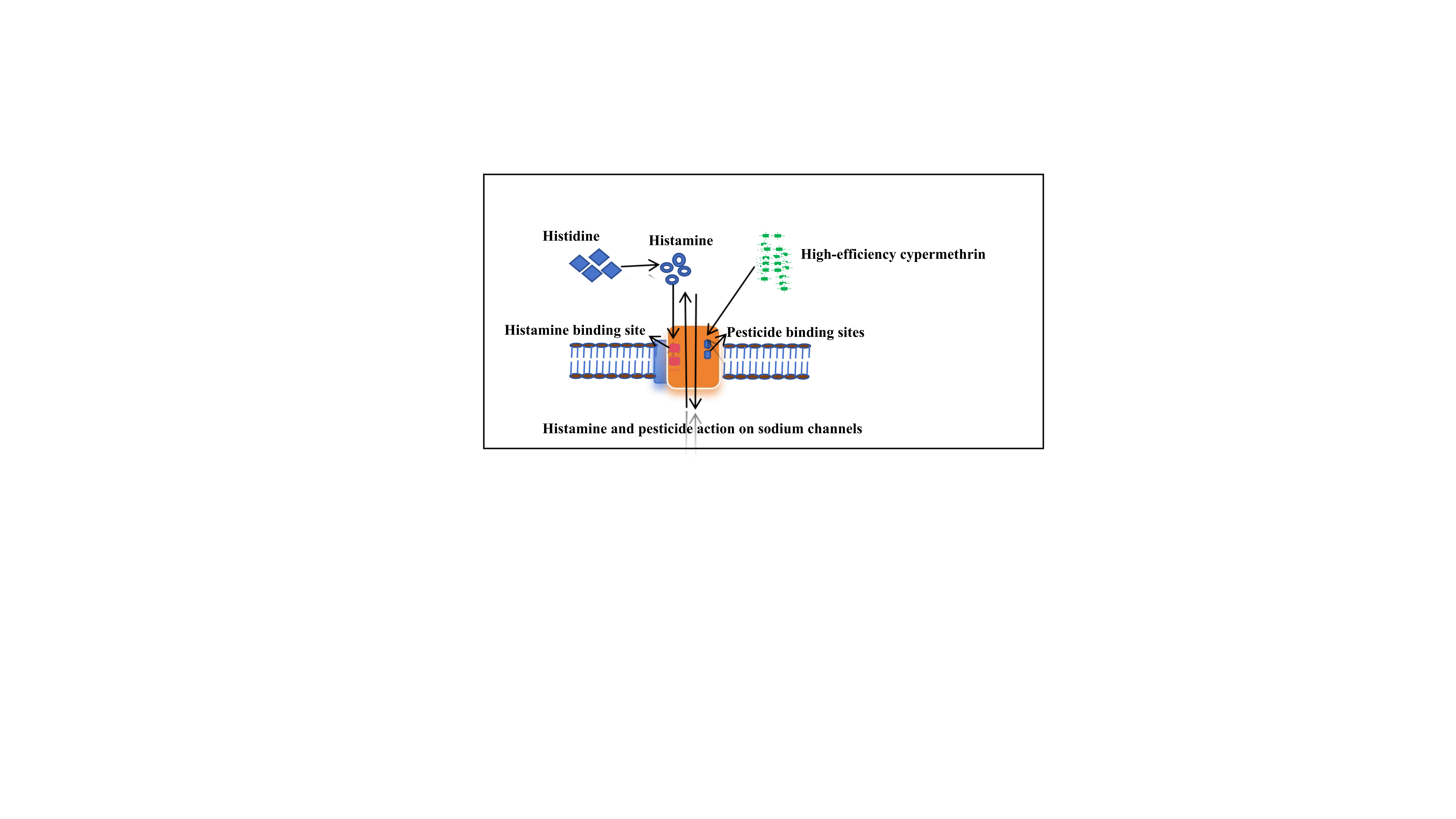
**

**Fig. S4.** Mechanisms of pesticide and histidine action on sodium channels.
